# The retromer and retriever systems are conserved and differentially expanded in parabasalids

**DOI:** 10.1242/jcs.261949

**Published:** 2024-07-12

**Authors:** Abhishek Prakash Shinde, Jitka Kučerová, Joel Bryan Dacks, Jan Tachezy

**Affiliations:** ^1^ Department of Parasitology, Faculty of Science, Charles University, BIOCEV, Průmyslová 595, 25242 Vestec, Czech Republic; ^2^ Division of Infectious Diseases, Department of Medicine and Department of Biological Sciences, University of Alberta, Edmonton, Alberta T6G 2G3, Canada; ^3^ Women and Children's Health Research Institute, University of Alberta, Edmonton, Alberta, Canada; ^4^ Centre for Life's Origins and Evolution, Department of Genetics, Evolution & Environment, University College London, Darwin Building, 99-105 Gower Street, WC1E 6BT, London, UK; ^5^ Institute of Parasitology, Biology Centre, Czech Academy of Sciences, 37005 České Budějovice (Budweis), Czech Republic

**Keywords:** Retromer, Retriever, Phylogenomics, Endomembrane, Evolution, Parabasalids

## Abstract

Early endosomes sort transmembrane cargo either for lysosomal degradation or retrieval to the plasma membrane or the Golgi complex. Endosomal retrieval in eukaryotes is governed by the anciently homologous retromer or retriever complexes. Each comprises a core tri-protein subcomplex, membrane-deformation proteins and interacting partner complexes, together retrieving a variety of known cargo proteins. *Trichomonas vaginalis*, a sexually transmitted human parasite, uses the endomembrane system for pathogenesis. It has massively and selectively expanded its endomembrane protein complement, the evolutionary path of which has been largely unexplored. Our molecular evolutionary study of retromer, retriever and associated machinery in parabasalids and its free-living sister lineage of *Anaeramoeba* demonstrates specific expansion of the retromer machinery, contrasting with the retriever components. We also observed partial loss of the Commander complex and sorting nexins in Parabasalia but complete retention in *Anaeramoeba*. Notably, we identified putative parabasalid sorting nexin analogs. Finally, we report the first retriever protein localization in a non-metazoan group along with retromer protein localization in *T. vaginalis*.

## Teaser

**Highlighted Article:** The retromer complex is selectively expanded in comparison to its anciently homologous retriever complex in the globally important parasite *Trichomonas vaginalis* and its relatives.

## INTRODUCTION

*Trichomonas vaginalis* is a parasitic flagellated protist that causes the commonly diagnosed non-viral sexually transmitted infection generally known as trichomoniasis. In 2020, 156 million new cases of this infection among people aged between 15 to 49 years were registered worldwide as surveyed by the World Health Organization (WHO). In women, a variety of symptoms are observed, such as vaginitis and cervicitis. Along with these symptoms, adverse outcomes include complications in pregnancy such as preterm birth, low birth weight, cervical cancer and a 1.5-fold higher risk of contracting an HIV infection (Edwards et al., 2016; McClelland et al., 2007). It is also reported that trichomoniasis infection during pregnancy can lead to intellectual disability in children (Mann et al., 2009). Although it is generally asymptomatic in men, it can cause urethral inflammation and increase the risk of prostate cancer (Sutcliffe et al., 2006; Krieger, 1995).

*T. vaginalis* is an anaerobic parasitic protist belonging to the monophyletic Metamonada group and Parabasalia subgroup (Burki et al., 2020; Adl et al., 2019). Parabasalids are flagellated organisms distinguished by the presence of hydrogenosomes, an anaerobic type of mitochondria, a cytoskeletal framework of unique microtubular elements known as the pelta-axostylar system, and a parabasal apparatus (Céza et al., 2022; Benchimol et al., 2000). Two distinct Parabasalia lineages, Trichomonadea and Tritrichomonadea, have primarily adopted a parasitic lifestyle (Malik et al., 2011). Recently, free-living *Anaeramoebae* were shown to represent the closest sister lineage of Parabasalia and found to serve a good contrast of lifestyles among related organisms (Stairs et al., 2021; Maciejowski et al., 2023).

*T. vaginalis* exhibits a massive genome expansion of selective gene families, including those of the membrane trafficking system involved in endocytosis and exocytosis from the cell. It also shows expansion of several of its secreted virulence factors (Carlton et al., 2007). The endomembrane machinery of *T. vaginalis* plays a crucial role in its pathogenesis. For instance, the lysosomal secretion of its virulence factors such as cysteine peptidases and cathepsin D is guided by the endosomal membrane trafficking system of the cell (Zimmann et al., 2022). To maintain this continuous secretion of virulence factors, endomembrane proteins responsible for their anterograde transport are expected to be rescued by retrograde trafficking to avoid their lysosomal degradation.

In eukaryotes, the membrane trafficking system constitutes anterograde and retrograde trafficking: anterograde trafficking transports cargoes from the endoplasmic reticulum through the trans-Golgi network (TGN) to the plasma membrane, whereas retrograde trafficking recycles the cargoes by sorting them in the early endosomes. This sorting of cargo in the endosomes helps with the recovery of essential resident endomembrane proteins that are required by the cell and used for new rounds of trafficking, while discarding the foreign material via lysosomal degradation through the endosomal sorting complexes required for transport (ESCRT) machinery (Raiborg and Stenmark, 2009; Pipaliya et al., 2021; Huotari and Helenius, 2011).

Endosomal retrograde trafficking was first reported to be carried out by a membrane coat complex, which was identified and characterized for the rescue of vacuolar protein-sorting receptor Vps10p, a sortilin homolog in yeast (Seaman et al., 1998). Vacuolar protein-sorting proteins Vps35p and Vps29p were shown to form a multimeric complex with Vps26p, Vps17p and Vps5p, which was collectively termed the retromer complex. The heterotrimeric complex of Vps35p, Vps29p and Vps26p conducts cargo selection for retrieval, whereas Vps5p and Vps17p assembly promotes vesicle formation via endomembrane deformation (Seaman et al., 1998). This was followed by the discovery of the VPS29, VPS35 and the two VPS26A and VPS26B (collectively VPS26A/B) homologs in humans (Edgar and Polak, 2000; Haft et al., 2000; Kerr et al., 2005). Indeed, the heterotrimeric subcomplex proteins of the retromer complex were confirmed to be highly conserved across pan-eukaryotic species (Koumandou et al., 2011).

In mammalian systems, the term retromer complex means only the conserved trimeric complex of VPS26, VPS29 and VPS35 (Seaman, 2021). In humans, the retromer complex was primarily shown to rescue cation-independent mannose 6-phosphate receptor (CIMPR), responsible for transporting lysosomal hydrolases possessing mannose 6-phosphate signals from the TGN to late endosomes (Seaman, 2004; Edgar and Polak, 2000; Arighi et al., 2004). Other than CIMPR and Vps10p, the retromer complex also rescues the late-Golgi enzyme Kex2 and a Golgi-resident endopeptidase, dipeptidyl amino peptidase (DPAP) (Seaman et al., 1998). Besides the retromer trimeric complex, TIP47 and adaptor proteins such as AP-1, AP-5 and epsinR are also shown to be critical for endosome to TGN transport of CIMPR (Díaz and Pfeffer, 1998; Meyer et al., 2000; Tu et al., 2020). Retromer complex genes are seen to be expanded in *T. vaginalis* in comparison with human and yeast (Carlton et al., 2007; Koumandou et al., 2011).

For a long time, the retromer complex was believed to be the only endosomal retrograde trafficking machinery. This changed with the recent discovery of the retriever complex in 2017, retromer-independent rescue machinery in the mammalian system (McNally et al., 2017). The retriever complex is also a heterotrimeric complex, structurally homologous to the retromer core complex. It consists of VPS26C, C16orf62 or VPS35-like (VPS35L) and the shared VPS29 (McNally et al., 2017; Chen et al., 2019). It selectively targets the sorting of plasma membrane proteins in a sequence-dependent manner. In mammalian cells, well-known cargoes for the retriever complex are the low-density lipoprotein receptors (LDLRs), LDLR-like protein (LRP-1) and α_5_β_1_-integrin (McNally et al., 2017; McNally and Cullen, 2018).

In addition to the core trimeric retromer and retriever complexes, the rescue of endomembrane proteins also depends on sorting nexins or cargo adaptors, which recruit the trimeric complexes to associate with the endomembrane cargo. They also mediate vesicle formation via endomembrane deformation. First identified in yeast assisting the retromer complex were the heterodimeric proteins Vps5p and Vps17p, both of which possess a Phox homology (PX) domain and a Bin, Amphiphysin and Rvs (BAR) domain (Nothwehr and Hindes, 1997; Teasdale and Collins, 2012; Lucas et al., 2016). Besides possessing a lipid-binding PX domain, the mammalian sorting nexins are categorized based on the presence or absence of additional domains: SNX-PX, SNX-BAR and SNX-FERM. All three categories of sorting nexins are known to assist the retromer complex. Of the SNX-FERM proteins, SNX17 and SNX31 are known to couple with the retriever complex (McNally et al., 2017; Harrison et al., 2014; Steinberg et al., 2013; Strochlic et al., 2007). However, no SNX proteins have yet been identified in *T. vaginalis* (Koumandou et al., 2011).

Among the accessory factors shared by both the rescue machineries, an essential one is the pentameric Wiskott–Aldrich syndrome protein and SCAR homolog (WASH) complex. This complex is responsible for actin branched network formation along the endosomal membrane to regulate endosomal tubule dynamics and endomembrane trafficking of the cargo (Derivery et al., 2009; Harbour et al., 2010). Apart from the WASH complex, the retriever machinery also requires another essential accessory factor, a huge complex of 13 proteins known as the CCC complex, comprising CCDC22, CCDC93, ten COMMD proteins and DENND10 (also known as FAM45A). Seeing as how the retriever and CCC complexes interact and depend on each other, both these complexes have been together termed as the Commander complex (Mallam and Marcotte, 2017; Healy et al., 2023).

Given the expansion of the retromer complex, the reported presence of the retriever component VPS35L in *T. vaginalis*, and the new availability of parabasalid genomes since the last time that these complexes were examined, we decided to undertake a molecular evolutionary analysis to determine the path that has produced this unusual protein complement. Moreover, as the endosomal system is involved in *T. vaginalis* pathogenesis and as the retriever complex has never been characterized outside of mammalian systems, we investigated the relative localization of retromer versus retriever components to determine whether there are multiple endosomal retrieval pathways.

## RESULTS

### Retromer complex genes are widely expanded in parabasalids and *Anaeramoeba* compared to retriever complex genes

**Fig. 1.**
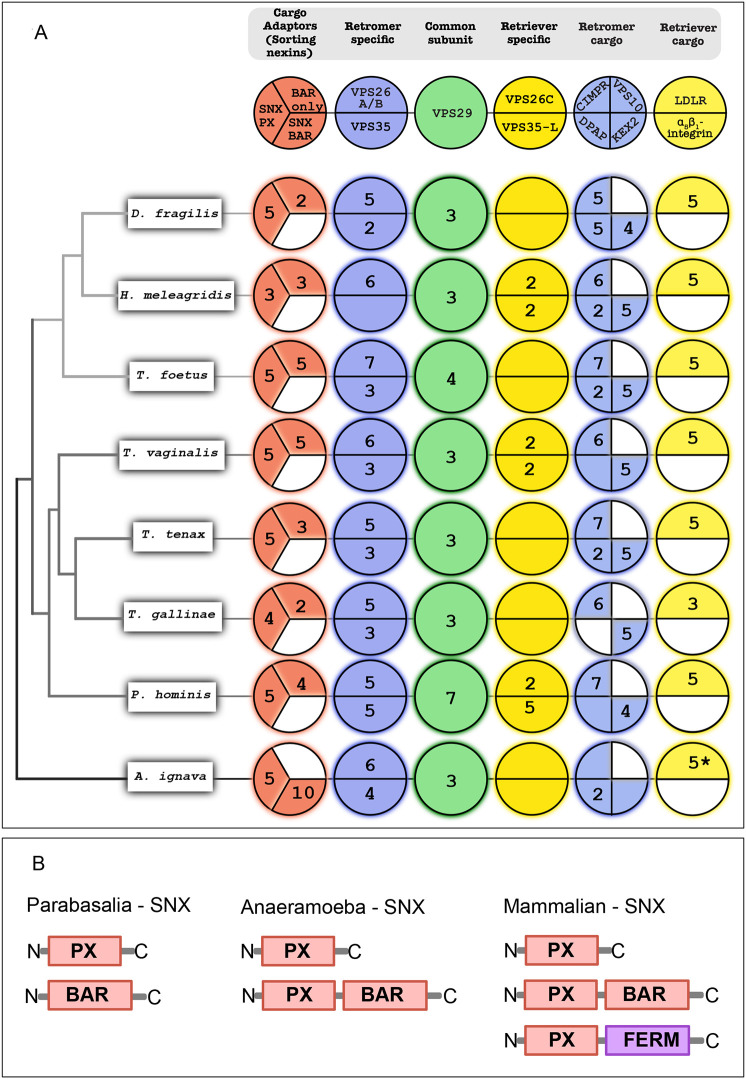
**Homology searches of endosomal retrieval complements and domain organization of sorting nexins.** (A) Coulson plot representing comparative genomics of the endosomal retrograde trafficking proteins and their specific known cargoes across selected Parabasalia organisms and *Anaeramoeba ignava*. Areas filled with color demonstrate positive occurrence of the protein homologs, numbers indicate the paralog count and colored sections with no numbers indicate a single paralog. White sections indicate the absence of protein homologs. Each plot column is annotated with the protein name at the top and the name of organism is mentioned on the left. The asterisk denotes the fact that the 5 LDLR paralogs were identified in *A. flamelloides* and not *A. ignava*. (B) Comparison of sorting nexins (SNX) identified in these organisms with the canonical mammalian SNX. Parabasalia contain SNX-PX and a unique SNX, homologous to the VPS5 C-terminus BAR domain. *Anaeramoeba* contain SNX-PX and SNX-BAR, whereas humans have an additional SNX-FERM.

In the previous studies conducted for the genomic survey of the retromer complex across pan-eukaryotic species, *T. vaginalis* was the only metamonad that showed multiple gene paralogs of trimeric retromer complex proteins: VPS26A/B, VPS29 and VPS35 (Koumandou et al., 2011). Pan-eukaryotic distribution of the retriever complex was limited only to VPS35L (McNally et al., 2017). Thus, we performed a deep-dive genomic analysis into the expansion of the retromer complex, while searching for the retriever complex and both their specific endomembrane cargoes in parasitic parabasalids and their closest free-living sister *Anaeramoeba ignava* (Fig. 1A).

In all the Parabasalia members, we observed an expansion of VPS26A/B ranging from five genes in *Trichomonas gallinae* and *Dientamoeba fragilis* to seven genes in *Tritrichomonas foetus*. Similarly, for VPS35, we observed the expansion ranging from two to five genes in nearly all parabasalids. The shared protein between the retromer and retriever complexes, VPS29, was seen to be expanded, ranging from three to four genes in all Parabasalia members, except *Pentatrichomonas hominis* that had seven genes*.* Somewhat unexpectedly, a similar pattern of gene expansions for the retromer complex proteins was also observed in the free-living *A. ignava* with six and four genes for VPS26A/B and VPS35, respectively, along with three genes for VPS29 (Fig. 1A).

A bigger surprise for us was seeing a far less expanded complement of the retriever complex-specific proteins VPS26C and VPS35L. In most parabasalids and *A. ignava*, only a single gene was found for both proteins. However, two copies were found in *Histomonas meleagridis* and *T. vaginalis*, whereas in *P. hominis*, two copies for VPS26C and five copies for VPS35L were identified.

**Fig. 2.**
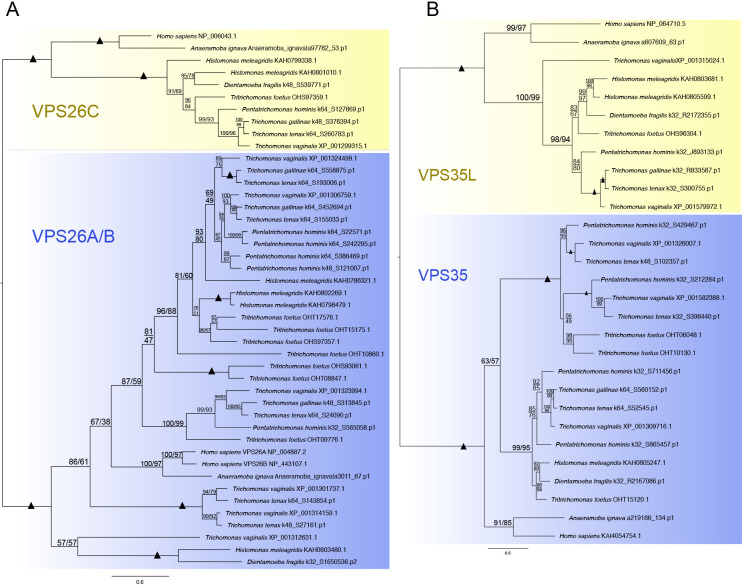
**Phylogenetic characterization of VPS26A/B with VPS26C and VPS35 with VPS35L.** (A) A maximum-likelihood phylogenetic analysis tree was constructed using IQTree (ModelFinder best fit: LG+F+G4) with 44 sequences and 222 sites for VPS26A/B and VPS26C. This tree includes all the orthologs of VPS26A/B and VPS26C identified in the comparative genomics for parabasalids. (B) A maximum-likelihood phylogenetic analysis tree was constructed using IQTree (ModelFinder best fit: Q.yeast+G4) with 29 sequences and 487 sites for VPS35 and VPS35L. This tree includes all the orthologs of VPS35 and VPS35L identified in the comparative genomics for parabasalids. *Homo sapiens* and *A. ignava* sequences were used as a reference for the analyses. The clades in blue depict the retromer complex proteins and the clades in yellow depict the retriever complex proteins. Support values for each node are depicted as ultrafast bootstrap values (UFB) on the left and non-parametric (NP) bootstrap values on the right (UFB/NP). Branches with support values of 100 for both ultrafast and NP bootstrapping are represented with black solid triangles.

VPS26C and VPS35L are ancient homologs of the retromer complex proteins VPS26A/B and VPS35, respectively, believed to be the result of ancestral duplication events before the last eukaryotic common ancestor (Koumandou et al., 2011; McNally et al., 2017). Therefore, we chose to validate our classifications via phylogenetics. This also allowed us to trace the origins of the observed expansions of the retromer components in parabasalids. We conducted phylogenetic analyses of the respective retromer and retriever complex homologs to investigate their classification in parabasalids and *A. ignava* (Fig. 2).

We conducted phylogenetic characterization of VPS26A/B with VPS26C (Fig. 2A), and characterization of VPS35 with VPS35L (Fig. 2B), including the human paralogs as outgroups and functionally verified markers. This analysis showed separate clades of VPS26A/B and VPS26C, confirming the classification of our homology searches in parabasalids (Fig. 2A). Similarly, VPS35 and VPS35L can also be seen to be classified in separate clades (Fig. 2B). This also confirms that VPS26C and VPS35L of the retriever complex have not been expanded. By contrast, VPS26 and VPS35 in parabasalids have been expanded via ancestral duplication events taking place prior to or in the last parabasalid common ancestor (LPCA).

**Fig. 3.**
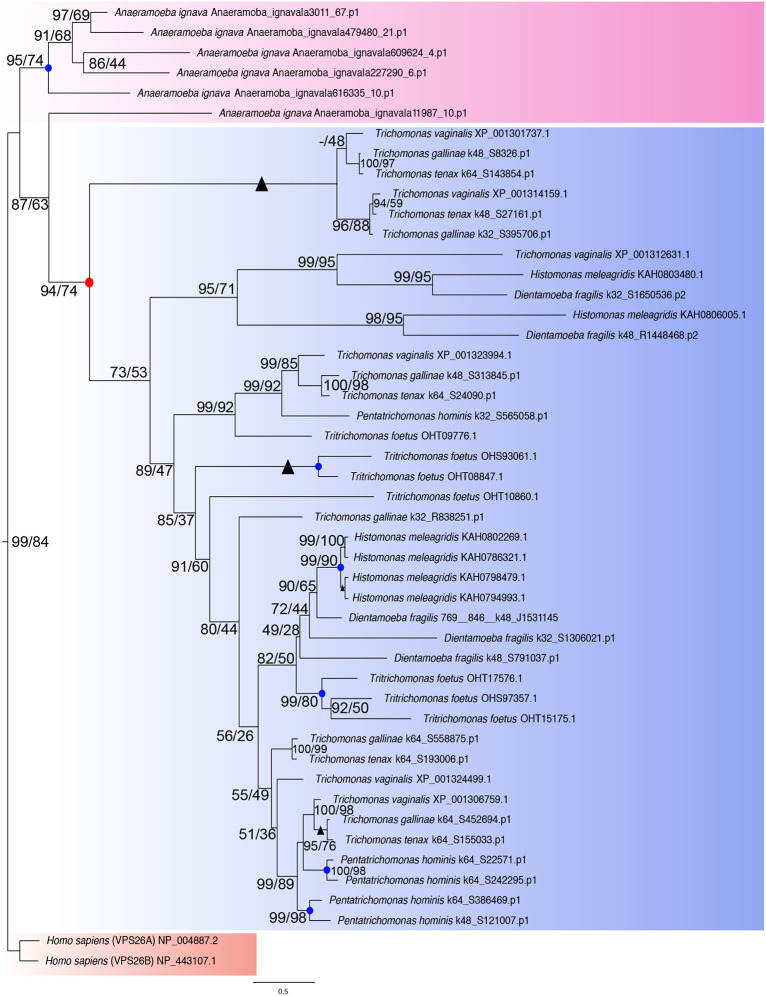
**Phylogenetic analysis of VPS26A/B paralogs identified across Parabasalia and *Anaeramoeba*.** A maximum-likelihood phylogenetic analysis tree was constructed using IQTree (ModelFinder best fit: Q.yeast+I+G4) with 48 sequences and 270 sites for VPS26A/B expansion events in the parasitic Parabasalia lineage and *A. ignava.* This includes all the orthologs identified from the homology searches*.* The clade in pink represents paralogs caused by species-specific expansion of VPS26A/B in *A. ignava*. The clade in blue represents orthologs of VPS26A/B identified in the parasitic Parabasalia lineage. VPS26A and VPS26B of *Homo sapiens* were used as an outgroup represented by a red clade. The red dot represents an ancestral duplication event in parasitic parabasalids, whereas the blue dots represent species-specific duplication events. Support values for each node are presented in the format of UFB/NP. Branches with support values of 100 for both ultrafast and NP bootstrapping are represented with black solid triangles.

We speculated from our homology searches that the expansion of the retromer complex in parabasalids can be timed for the duplication events and possibly traced back to *A. ignava*. Hence, we conducted phylogenetic analyses for each retromer complex protein to trace its expansion in *A. ignava* and the parabasalids (Fig. 3). For *A. ignava*, we carefully selected the sequences that showed conserved sequence homology and ignored the partial sequences. All the *A. ignava* hits for VPS26A/B demonstrated sequence homology, whereas only two hits of VPS29 and VPS35 each showed the complete presence of sequence homology. All the sequences from parabasalid searches were included in the analyses.

In the phylogenetic analysis of VPS26A/B (Fig. 3), we used human VPS26A and VPS26B as an outgroup. We observed that the expansion of VPS26A/B in *A. ignava* was independent of the expansion observed in parabasalid members. Among parabasalids, the expansion of VPS26A/B appears to be a result of numerous ancestral duplication events. Additionally, species-specific duplication events appear to have occurred in *T. foetus*, *H. meleagridis* and *P. hominis*.

After confirming the lineage-specific expansion of the retromer complex in parabasalids and *A. ignava* for VPS26, we decided to use the *A. ignava* clade as an outgroup for the other analyses to increase robustness. We conducted phylogenetic analysis for the expansion of VPS29 and VPS35 of retromer complex in parabasalids (Figs S1 and S2). We show an ancestral duplication event in LPCA resulting in two parabasalid-specific homologs for both the proteins, i.e. VPS29A/B and VPS35A/B. Similar to the expansion of VPS26A/B, we observed species-specific duplication events in *P. hominis* and *T. foetus* for VPS29 and VPS35.

### Parabasalids encode retromer and retriever complex-specific cargo proteins

As the retromer complex proteins were seen to be expanded alongside contrasting observations for the retriever complex-specific proteins in Parabasalia members and *A. ignava*, we extended our searches to the specific endosomal membrane cargo proteins for both these machineries (Fig. 1A). For the endomembrane cargoes of the retromer complex, we looked for CIMPR, Vps10p (VPS10), DPAP and KEX2*.* Consistent expansion was observed for CIMPR, with five to seven genes in all the parasitic parabasalids; however, only a single gene of putative CIMPR was found in the free-living *A. ignava.* Similarly, KEX2 was also expanded with four to five genes in all parabasalids, whereas *A. ignava* only had a single gene. Expansion of DPAP was observed in all the Tritrichomonadea ranging from two to five genes, whereas a single gene was found in *T. vaginalis* and *P. hominis*, two genes in *Trichomonas tenax* and *A. ignava*, and none in *T. gallinae*. VPS10 was seen to be completely missing in all taxa searched.

Next, we chose two functionally verified retriever complex cargo candidates: LDLR and α_5_β_1_-integrin. Although LDLR is known to be present in non-metazoan species, α_5_β_1_-integrin is thus far unique to humans; however, integrins have been identified within Amorphea (McNally et al., 2017; Kang et al., 2021). Contrasting to the lack of expansion of retriever machinery components, three to five genes for LDLR were found in all the parabasalids. However, LDLR was seen to be completely absent in *A. ignava* but, alternatively, five genes were found in *Anaeramoeba flamelloides* of the *Anaeramoeba* lineage. This could have been because of the fragmented datasets of *A. ignava* (Maciejowski et al., 2023). α_5_β_1_-integrin proteins were seen to be completely missing in both parabasalids and *Anaeramoeba*. These results suggest a similar pattern of evolution for both the rescue machineries in parasitic parabasalids and *Anaeramoeba* regardless of their contrasting lifestyles.

### The evolution of the WASH complex and the CCC complex is consistent with the evolution of the retriever complex in Parabasalia and *Anaeramoeba* lineages

**Fig. 4.**
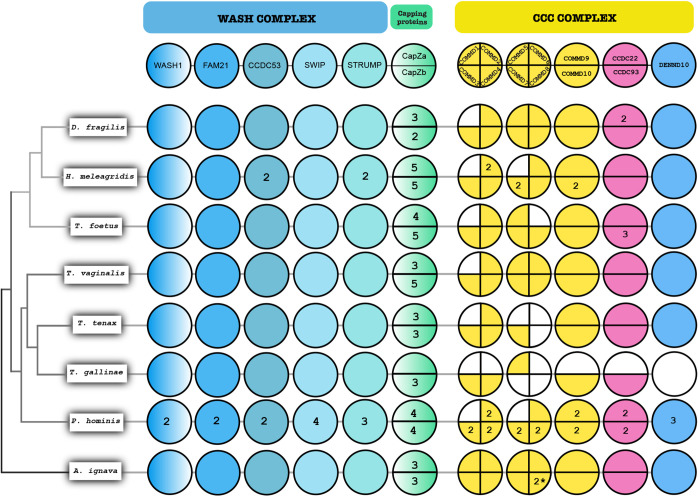
**Coulson plot representing comparative genomics of the WASH complex and capping proteins shared by retromer and retriever complexes, and the CCC complex for the retriever complex in selected parabasalid species and *A. ignava*.** Areas filled with color demonstrate positive occurrence of the protein homologs, numbers indicate the paralog count and colored sections with no numbers indicate a single paralog. Each plot column is annotated with the protein name at the top and the name of organism is mentioned on the left. The asterisk denotes that two paralogs of COMMD8 were identified in *A. flamelloides* and not in *A. ignava*.

The pentameric WASH complex regulating the endosomal tubule dynamics is known to be shared by the retromer and retriever complexes and it plays a crucial role in the successful rescue of endomembrane cargoes by both these machineries. We conducted a comparative genomic survey of the WASH complex in Parabasalia and *A. ignava* genomes to understand the consistency of its evolutionary pattern with either of the heterotrimeric complexes (Fig. 4).

Based on our previous observation of the shared VPS29 protein being expanded like the retromer complex in both Parabasalia and *Anaeramoeba* lineages (Fig. 1A; Fig. S1), we expected the WASH complex to showcase a similar pattern of evolution. However, to our surprise, we observed only a single gene homolog of the WASH1 protein in nearly all the parabasalids and *A. ignava*. We observed the same pattern of evolution for FAM21 (WASHC2) as well, which is known to interact via its tail with VPS35 of the retromer complex and the CCDC93–CCDC22 dimer of the Commander assembly with the retriever complex (Jia et al., 2012; Simonetti and Cullen, 2019). Similarly, CCDC53 (WASHC3) is also present as a single gene in five of the Parabasalia species and *A. ignava*; however, two paralogs were seen in *H. meleagridis* and *P. hominis* each. A single gene of SWIP (WASHC4) was seen in nearly all the parabasalids and *A. ignava*. SWIP was also recently identified to mediate the recruitment of the WASH complex to the endomembrane, independent of the retromer complex (Dostál et al., 2023). The final WASH complex component, strumpellin or STRUMP (WASHC5), also demonstrates a similar fashion of evolution, with single genes identified in the majority of the parabasalids and *A. ignava*. Like CCDC53, STRUMP is also present as two and three paralogs in *H. meleagridis* and *P. hominis*, respectively. Hence, the WASH complex follows a similar pattern of evolution to the retriever complex. We also confirmed species-specific duplication events specifically in *H. meleagridis* and *P. hominis* by phylogenetic analysis of the WASH complex proteins in Parabasalia members (Fig. S3).

Additionally, the dimeric capping proteins CapZα and CapZβ, known to associate with the WASH complex and promote endosomal maturation (Wang et al., 2021), were also investigated. We observed an expansion of CapZα ranging from three genes in *D. fragilis*, *T. vaginalis*, *T. tenax* and *A. ignava* to five genes in *H. meleagridis.* Four genes were seen in *T. foetus* and *P. hominis.* Only a single gene was identified in *T. gallinae*. For CapZβ, we observed an expansion ranging from two genes in *D. fragilis* to five genes in *H. meleagridis*, *T. foetus* and *T. vaginalis*. An expansion of three genes was seen in *T. tenax*, *T. gallinae* and *A. ignava* and an expansion of four genes was seen in *P. hominis*.

Finally, we investigated the evolutionary pattern of the CCC complex (Fig. 4), consisting of 13 proteins, involving ten COMMD proteins, CCDC22, CCDC93 and DENND10 proteins exclusively interacting with only the retriever complex and forming a megacomplex known as the Commander assembly. The CCC complex plays a crucial role in the retriever machinery by forming a link between the retriever complex and the WASH complex to mediate actin polymerization (Simonetti and Cullen, 2019; Healy et al., 2023).

We observed that the *Anaeramoeba* lineage shows the presence of all the CCC complex proteins, whereas COMMD1 of the CCC complex was seen to be completely lost in all the parasitic parabasalid members. We found single gene homologs of all the CCC complex proteins in the *Anaeramoeba* lineage (surveyed in *A. ignava* or *A. flamelloides*) and in nearly all the parabasalids. However, we found an expansion of two genes for all the identified CCC complex proteins in *P. hominis* except for three paralogs identified for DENND10 and the presence of a single gene of COMMD4. A similar pattern of expansion was observed in *H. meleagridis* too; an expansion of two genes was seen for COMMD2, COMMD7 and COMMD10, and the rest of the CCC complex proteins identified are present as single homologs. Most of the expansions of COMMD proteins seen in *H. meleagridis* and *P. hominis*, such as the WASH complex, appear to be a result of species-specific duplication events (Fig. S4).

In all other parabasalids, the CCC complex proteins majorly contain single genes for each protein identified in our genomic survey while showing a complete loss of COMMD1. *T. tenax* appears to have lost COMMD5, COMMD6 and COMMD8. *T. gallinae* was seen to be missing COMMD1, COMMD2, COMMD6, COMMD7, COMMD8 and COMMD9, along with CCDC22 and DENND10, which could have been a result of its poorer BUSCO score (Maciejowski et al., 2023). Among Tritrichomonadea, *T. foetus* appears to have lost COMMD4 along with COMMD1, while containing single homologs of other CCC complex proteins except for an expansion of three genes for CCDC93. *D. fragilis* is seen to have a single gene homolog for all the CCC complex proteins except for the expansion of two genes for CCDC22 and, like other parabasalids, it is seen to have lost the COMMD1 protein. A phylogenetic characterization of all the COMMD proteins identified in the *Anaeramoeba* and Parabasalia members was conducted using human proteins as verified markers (Fig. S4).

### A possible answer to the mystery of the missing sorting nexins in the Parabasalia lineage

Both the retromer and retriever complex machineries use sorting nexins to recruit the trimeric complexes at the endosomal membrane for its interaction with the endomembrane cargo proteins to be recycled, while also promoting the deformation of the membrane for vesicle formation (Gallon and Cullen, 2015). In mammals, SNX1, SNX2, SNX5 and SNX6 of the SNX-BAR category with the PX and BAR domain assist the retromer complex (Collins et al., 2005; Shi et al., 2006). Recently, SNX3 of the SNX-PX category was confirmed as a functional sorting nexin of the retromer complex assembly with only a PX domain (Leneva et al., 2021; Harterink et al., 2011; McGough et al., 2018; Strochlic et al., 2007). SNX27 and SNX17 are examples of SNX-FERM sorting nexins associated with retromer assembly and retriever assembly, respectively (Chandra et al., 2021; Healy et al., 2023; Gallon et al., 2014). Thus, it was puzzling that no such SNX proteins were identified in prior analyses (Koumandou et al., 2011; Gallon and Cullen, 2015). To investigate this mystery, we decided to explore this in all the parabasalids and *A. ignava* to find sorting nexins using comparative genomics (Fig. 1A).

We found that in *A. ignava*, the canonical sorting nexins, SNX-BAR proteins, have ten paralogs, whereas there are five paralogs of SNX-PX proteins. We found SNX-FERM proteins to be completely absent in both the Parabasalia and *Anaeramoeba* lineages. In all the Parabasalia members, we observed three to five genes of SNX-PX proteins; however, SNX-BAR proteins were seen to be completely absent. Additionally, we also found two to five genes of ‘BAR-only’ proteins in all the parabasalids, which are missing in the *Anaeramoeba* lineage. BAR-only proteins represent homology toward the C-terminus BAR domain of the fungal VPS5 (Fig. 1B). Upon critically looking into the genome assembly of *T. vaginalis*, the SNX-PX homologs did not appear to be in the vicinity of BAR-only homologs and are also located on different chromosomes. This suggests that the SNX-PX and BAR-only proteins in parabasalids are independent proteins and possibly not a result of fragmentation in the genome. We also attempted phylogenetic analysis for the identified SNX proteins. Unfortunately, such a tree showed no resolution. Thus, we have identified putative candidates for sorting nexins that could help mitigate the loss of standard eukaryotic sorting nexins in parasitic parabasalids.

### *T. vaginalis* retromer and retriever components are expressed and are modelled to have conserved structures to those of human counterparts

**Fig. 5.**
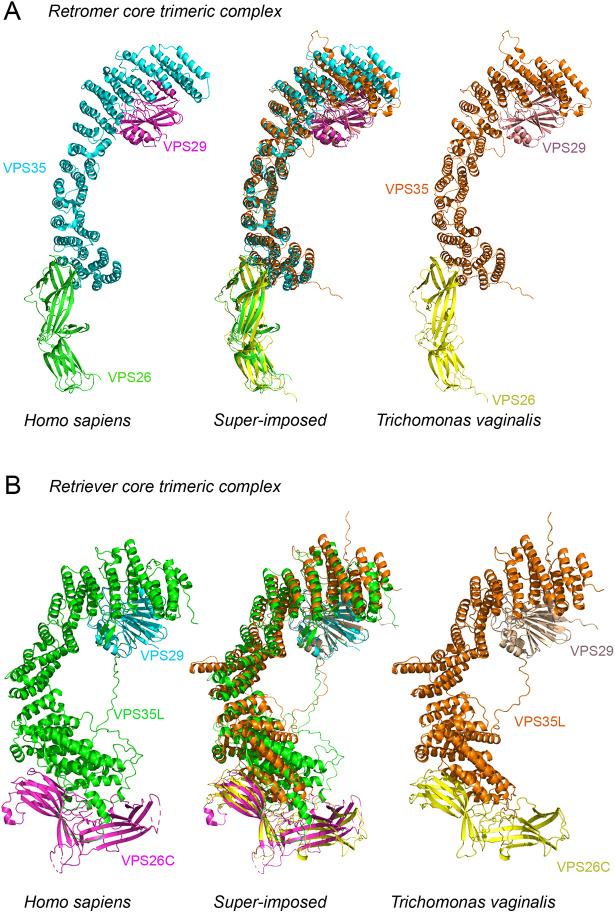
**Molecular modelling of *Trichomonas vaginalis* retromer and retriever complexes compared to human crystal structures.** Retromer (A) and retriever (B) trimeric core complexes predicted in *T. vaginalis* based on known structural models of retromer (PDB: 6H7W) and retriever (PDB: 8SYN) in humans. Each protein is labelled and color coded to the structural models of retromer and retriever proteins. Structure models of each *T. vaginalis* retromer and retriever protein were chosen based on the highest sequence similarity of the paralog with human protein sequences (see Fig. S5). Superimposed structural alignments for each trimeric complex are labelled as such.

In order to better understand the *T. vaginalis* retromer and retriever proteins that we identified, we delved into publicly available gene expression data and tested the structural homology of both the trimeric complexes in comparison with the known structures of human retrieval trimeric complexes (Fig. 5). As summarized in Table S8, we found evidence for gene expression for all *T. vaginalis* proteins that we identified. Moreover, we found many of the respective proteins in a proteomic study of *T. vaginalis* lysosomes, further confirming our assessment of this expanded complement as valid separate encoded genes and not informatic mis-predictions. Notably, in mammalian systems, the retromer components VPS35 and VPS26A/B are more highly expressed than their retriever paralogs, VPS35L and VPS26C, respectively. We found a similar trend in the two transcriptomic datasets that we surveyed. In all but one instance, the most highly expressed paralog of each retromer component was approximately two to five times more highly expressed than the equivalent retriever component (Table S8).

For structural comparisons, the most highly expressed paralog of each component for the retromer and retriever core trimeric complexes was used. This was based on a transcriptomic analysis conducted on *T. vaginalis* under anaerobic conditions (Gould et al., 2013) and identification of the paralogs by proteomic analysis (Zimmann et al., 2022) (Table S8). Tertiary structures of the predicted *T. vaginalis* retromer and retriever proteins were aligned with the known human crystal structures (Kovtun et al., 2018; Boesch et al., 2023). All the structural alignments conducted showed high confidence with a template modelling (TM)-score of at least 0.79, indicating the presence of aligned residues in about the same fold of the protein structures (Table S9). A three-dimensional (3D) super-imposition of human and *T. vaginalis* retromer complex shows homologous structures in both the divergent eukaryotes (Fig. 5A). Similarly, a 3D super-imposition of the human and *T. vaginalis* retriever complex was conducted, which too demonstrated structural homology (Fig. 5B).

We also predicted the secondary structures of the same paralogs of the *T. vaginalis* retromer and retriever complexes and aligned them to all the paralogs identified for each of the proteins, along with the human protein sequences to identify the conservation of functional residues (Collins et al., 2008; Damen et al., 2006; Kendall et al., 2022; Gallon et al., 2014). Among all identified protein paralogs of retromer and retriever complex proteins in *T. vaginalis*, multiple sequence alignments did demonstrate mutations in some paralogs leading to substitution or elimination of essential amino acid residues in the conserved regions (Fig. S5). Notably, among all VPS26A/B paralogs of *T. vaginalis*, the SNX27 PDZ domain-interacting residues are lost, which correlated with absence of SNX27 across all parabasalids. Meanwhile, other functional residues are mostly conserved, with site mutations in a few paralogs for the retromer and retriever proteins (Fig. S5).

### Retromer and retriever complexes localize differently from one another in *T. vaginalis* cells

**Fig. 6.**
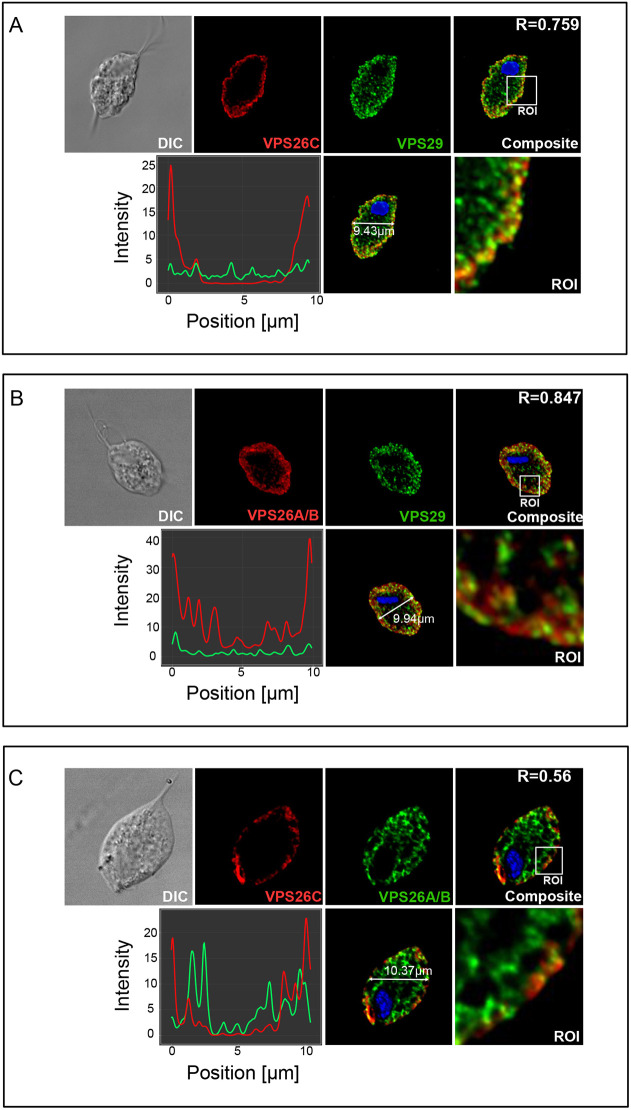
**Differential cellular localization of the retromer and retriever complexes in *Trichomonas vaginalis*.** (A) Colocalization of the retriever-specific VPS26C with the common subunit VPS29. *Z*-stack composite and channel *z*-stacks of immunofluorescence confocal microscopy images of *T. vaginalis* cells labelled with HA–HA–VPS26C (red) and V5–VPS29 (green), as well as the cell position versus channel intensity plot, cell composite with scale and an enlarged region of interest (ROI) are shown. (B) Colocalization of the retromer-specific VPS26A/B and shared subunit VPS29. Immunofluorescence confocal microscopy images of *T. vaginalis* cell labelled with HA–HA–VPS26A/B (red) and V5–VPS29 (green) as well as the cell position versus channel intensity plot, cell composite with scale and enlarged ROI are shown. (C) Colocalization of the retromer and retriever complexes. Immunofluorescence confocal microscopy images of *T. vaginalis* cell labelled with HA–HA–VPS26C (red) and V5–VPS6A/B (green), as well as the cell position versus channel intensity plot, cell composite with scale and enlarged ROI are shown. Images are representative of 10 or more cells.

The retriever complex has only been characterized by molecular cell biological techniques in mammalian systems. Therefore, we decided to test whether these complexes act similarly in *T. vaginalis* as in mammals. We analyzed the subcellular localization of retromer and retriever complexes to distinguish trafficking pathways using immunofluorescence confocal microscopy (Fig. 6). Although in humans, the retromer and retriever proteins are known to colocalize at the same endosomal retrieval subdomains (McNally et al., 2017), the retromer complex recycles its cargo to the TGN and the plasma membrane, whereas the retriever complex recycles its cargo only to the plasma membrane. We hypothesized that this phenomenon is conserved in *T. vaginalis.*

We co-expressed the retriever complex-specific protein VPS26C, tagged with a double human influenza hemagglutinin (HA) tag, alongside the shared protein between retromer and retriever complexes, VPS29, tagged with a V5 tag (Fig. 6A). We observed VPS26C to be distributed along the cell membrane (red channel). Although VPS29 (green channel) colocalized significantly with VPS26C on the cell surface, it was also observed in internal vesicles. The composite image confirms the colocalization of VPS26C with VPS29 as can also be seen in the region of interest (ROI) image (Fig. 6A). The represented image showed the colocalization parameter, the Pearson co-efficient (R-value), to be 0.759 for the complete cell.

Next, we studied the colocalization of VPS26A/B of the retromer complex with the shared VPS29 protein. As above, VPS29 was tagged with V5 and VPS26A/B was tagged with a double HA tag (Fig. 6B). We observed VPS26A/B to be distributed along the cell membrane as well as inside the cells on the internal vesicles (red channel). Similarly, VPS29 (green channel) was also localized on the cell membrane and internal vesicles. From the composite image and the ROI, VPS26A/B and VPS29 colocalize internally on the membranes of vesicles, most arguably, endosomes, along with their distribution on the cell membrane (Fig. 6B). The represented image showed an R-value of 0.874 for the complete cell.

Finally, we co-expressed V5-tagged VPS26A/B of the retromer complex with HA-tagged VPS26C of the retriever complex (Fig. 6C). As expected, we observed VPS26C (red channel) to be localized abundantly along the plasma membrane, whereas VPS26A/B (green channel) was seen to be localized internally as well as towards the plasma membrane; both these localizations were consistent with Fig. 6A,B. As can be seen from the composite and ROI images, the retromer and retriever components appear to colocalize only in certain spots on the plasma membrane (Fig. 6C), significantly less than the membrane colocalization of VPS26C with the shared VPS29 protein (Fig. 6A). The represented image showed an R-value of 0.56 for the complete cell. We also provide intensity plots for each colocalization analysis determining the channel intensities in different cellular locations (Fig. 6A–C), clearly showing the retromer complex being formed at the cell surface as well as internally. In contrast, the retriever complex was formed only on the cell surface while also sharing some localization in the cell, possibly on shared endosomal retrieval subdomains.

## DISCUSSION

Among the previous studies conducted on the retromer complex, only one pan-eukaryotic comparative genomic survey has reported the presence of an endomembrane rescue machinery in parabasalids. This showed a unique expansion of all the trimeric retromer complex proteins in *T. vaginalis*, whereas other Metamonada species with the presence of retromer complex lack this expanded complement (Koumandou et al., 2011). Despite its essential role in the membrane trafficking system in eukaryotes, more than a decade later, there has still been no further investigation to study such a peculiar phenomenon in this significant human parasite. Our studies provide the first comprehensive investigation of the complete endomembrane rescue machinery in not only *T. vaginalis*, but also in all the other parasitic parabasalids and their free-living sister of the *Anaeramoeba* lineage.

Our investigations provide the first evidence for the presence of the retriever complex machinery in Parabasalia and *Anaeramoeba* (other than *T. vaginalis*). To assess whether this complex was encoded and expanded as with retromer, we conducted our genomic survey in all the available parasitic parabasalids and *Anaeramoeba*. To our surprise, the VPS26C and VPS35L proteins of the retriever complex did not appear to be expanded as the retromer complex proteins VPS26A/B and VPS35. This contrast in evolutionary pattern was found in *A. ignava* alongside all the parasitic parabasalids. The VPS29 protein shared between both these trimeric complexes is seen to be expanded similar to VPS26A/B and VPS35 of the retromer complex in all the investigated taxa. Based on this, the selective forces acting to cause expansion of the retromer complex appear also to be acting on VPS29.

Based on our phylogenetic analyses to study the expansion of the retromer complex (Figs S1 and S2), we also propose that due to a duplication event in the LPCA, VPS29 and VPS35 evolved in parabasalids with two orthologs each (VPS29A/B and VPS35A/B) in the parasitic parabasalids. Meanwhile, a more recent duplication event in the evolution of VPS26A/B appears to have occurred only in Tritrichomonadea, whereas its expansion in Trichomonadea of the Parabasalia lineage appears to be a result of ancestral duplication events, except for *P. hominis*.

The CCC complex is also largely unexpanded in parabasalids, as with VPS26C and VPS35L. This suggests a synchronized evolution of both these interacting complexes in the retriever-mediated rescue machinery. Consistent with this, the CCC complex is also absent in the fungal lineage and suggests that it does not interact with the retromer assembly (McNally et al., 2017; Healy et al., 2023). Another shared component between the retromer and the retriever complex that we subjected to genomic survey is the pentameric WASH complex, which is responsible for actin polymerization forming the endomembrane tubules and endomembrane maturation, making it highly essential to the function of both the complexes. Due to its association with the retriever as well as the retromer complex, we expected its pattern of evolution to resonate with that of the shared VPS29 protein. However, to our surprise, we saw that the evolution of the WASH complex is like that of the retriever complex in the Parabasalia and *Anaeramoeba* lineages. It also critically differs from the pan-eukaryotic presence of the retromer complex and demonstrates a distribution similar to that of the retriever complex, while being absent in the fungal lineage (McNally et al., 2017). Based on our investigations, we suggest that the distinct evolution trajectories of the retromer and retriever complexes could have driven the evolutionary patterns of these conserved accessory components. However, the difference in the evolution of shared component VPS29 and the WASH complex remains to be assessed.

We also provide evidence suggestive of the presence of putative sorting nexins in parabasalids and *Anaeramoeba*, previously thought to be lost in *T. vaginalis*. Very recently, it was confirmed that SNX3, a sorting nexin containing only the PX domain, is a functional cargo adaptor for the retromer complex, independently capable of retromer-mediated cargo rescue (Cui et al., 2019; Strochlic et al., 2007). Based on this information and our genomic survey, we hypothesize that the putative SNX-PX homologs identified in parabasalids are highly divergent sorting nexins acting as the functional cargo adaptors for both the retromer and retriever complexes (Fig. 1). We hope that further studies and deeper investigations will evaluate and validate our hypothesis.

An interesting observation for retromer versus retriever protein expression levels in various transcriptomic analyses in different growth conditions of *T. vaginalis* consistently showed retromer proteins to be quantitatively more highly expressed in comparison to retriever complex proteins (Gould et al., 2013; Margarita et al., 2022; Cheng et al., 2015; Huang et al., 2014; 2015, 2021; Xie et al., 2023). This is consistent with the trend observed in humans (Itzhak et al., 2016). Our investigations with the sequence alignments for the paralogs of each analyzed protein in *T. vaginalis* with their human homologs did identify subtle differences in the functional residues of the protein. This suggests that multiple paralogs of each core retrieval trafficking protein might perform differentially under varying conditions of cell growth or could represent differential cargo preference. Based on the 3D structural superimposition of *T. vaginalis* retromer and retriever trimers with the respective human trimeric structures, we can hypothesize that the structural homology of the retrieval trimers with the human protein complex suggests a functional homology (Fig. 5).

**Fig. 7.**
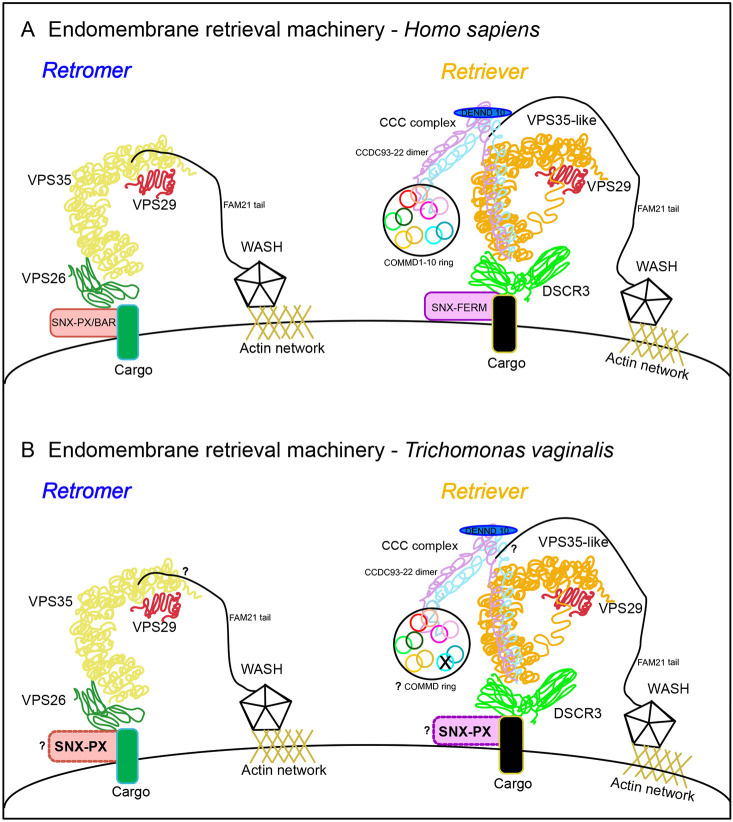
**Endosomal retrieval assembly hypothesis.** (A) Cartoon depiction of the canonical retromer complex and retriever complex machineries on the sorting endosomal membrane of a mammalian system, ideally, *H. sapiens*. Canonically, sorting nexins in the retromer complex have an additional BAR domain and the sorting nexins for the retriever complex sorting have an additional FERM domain, as labelled. (B) Cartoon depiction of the reconstruction of the retromer and retriever complex machineries in the parasitic parabasalid system, ideally, *T. vaginalis*. The missing COMMD1 of the CCC complex is marked with an ‘X’. Sorting nexins are proposed to be SNX-PX for both the complexes. Unknown interactions in *T. vaginalis* but known in human are marked with question marks.

The retromer and retriever complexes demonstrate different subcellular localization patterns in the model parasitic parabasalid *T. vaginalis*. Having confirmed the distinct evolutionary patterns for the retromer and retriever complex machineries, we evaluated whether they differed in their rescue pathways in *T. vaginalis* (Fig. 6). Based on our observations, we suggest that the retromer complex rescues its specific endomembrane cargoes either back to the cell surface or internally to the TGN, whereas the retriever complex rescues its endomembrane cargoes primarily to the cell surface. This confirms that both these complexes demonstrate functional homology in this parasitic protist lineage. However, in Fig. 6C, the colocalization values for VPS26A/B and VPS26C hint towards the possibility for them to share endosomal retrieval subdomains in the *T. vaginalis* cells like in the human cells (McNally et al., 2017). This is the first report of a cellular localization study and functional conservation of the retromer complex in any metamonad species and the first non-metazoan report for cellular localization of the retriever complex. Based on all these findings, we attempted reconstruction of both the retrieval complex assemblies on the endosomal membrane of *T. vaginalis* (Fig. 7)*.*

**Fig. 8.**
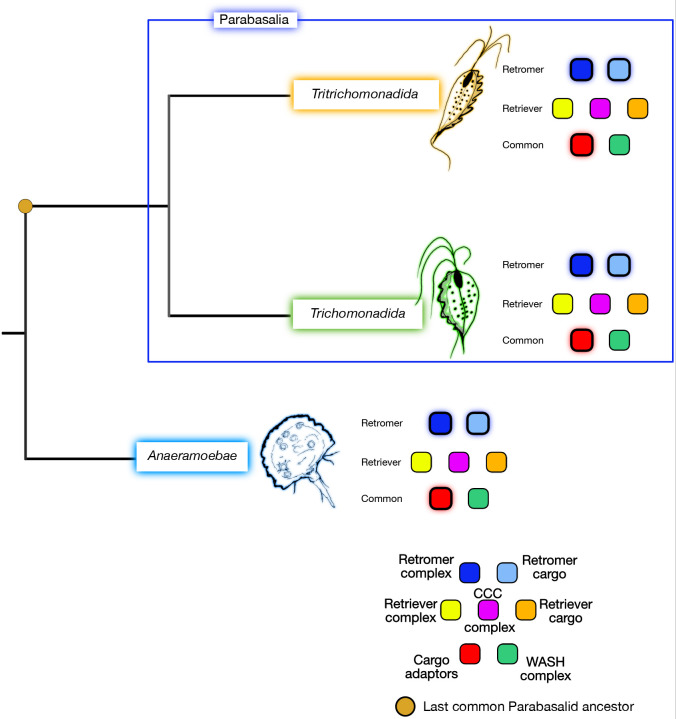
**Conclusion of phylogenomics in Parabasalia and *Anaeramoeba* lineages.** The outer glows of the colored boxes represent gene expansion of the proteins belonging to specific complexes.

In conclusion, we provide a comprehensive investigation of the endomembrane rescue machinery in parasitic parabasalids and their free-living sister *Anaeramoeba*. It reveals the distinct evolution of the retriever complex from the retromer complex, with the shared VPS29 protein exhibiting an expanded evolutionary pattern like that of the retromer complex and the shared WASH complex, and the retriever-specific CCC complex exhibiting an evolutionary pattern like that of the retriever complex. Additionally, the presence of putative sorting nexins in parabasalids and *Anaeramoeba* challenges previous beliefs (Fig. 8). The study also demonstrates different functional pathways for the retromer and retriever complexes in *T. vaginalis*. These findings contribute to our understanding of the evolutionary dynamics and functional conservation of both the rescue machineries in parasitic protists, and we hope that further investigations in other parasitic systems will evaluate and validate our hypothesis.

## MATERIALS AND METHODS

### Taxa genomic and transcriptome dataset acquisition

Genomes, protein datasets, and transcriptomes were acquired from the National Centre for Biotechnology Information (NCBI) for *T. vaginalis* (Carlton et al., 2007); *T. foetus* (Benchimol et al., 2017); *H. meleagridis* (Palmieri et al., 2021); and *D. fragilis*, *P. hominis*, *T. tenax*, and *T. gallinae* (Handrich et al., 2019). *A. ignava* and *A. flamelloides* transcriptome data were obtained from FigShare (Stairs et al., 2021). The list and sources of the datasets are provided in Table S1.

### Homology searches and domain analysis

Homology searches of the components in endomembrane retrograde transport machineries and their cargoes were conducted using Analysis of Molecular Evolution with BAtch Entry (AMOEBAE) (Barlow et al., 2023). *Homo sapiens* and *Saccharomyces cerevisiae* protein sequences from NCBI were used as queries for the initial homology searches. Forward searching was performed with BLASTp and tBLASTn, and the e-value maximum limit parameter was set to 0.05 to sort all the positive hits. AMOEBAE then confirmed the positive hits by performing reciprocal BLAST searches with the same e-value cutoff. Positive hits from this were used to prepare hidden Markov models (HMMs) to conduct HMMER searches using AMOEBAE for all the queries across the above obtained Parabasalia and *Anaeramoebae* genome and transcriptome datasets.

The final positive hits were validated and confirmed for homology using HHPred (Zimmermann et al., 2018) and InterProScan (Paysan-Lafosse et al., 2023) for the domain presence. The confirmed positive hits with accession numbers of the proteins are listed in Tables S2–S6.

### Phylogenetic analyses and tree construction

All the protein sequence alignments were created using MAFFT v7.505 (Katoh et al., 2019). For visualization of the alignments, a fast and lightweight software, AliView, was used (Larsson, 2014). The alignments were trimmed to obtain the informative regions using Block Mapping and Gathering with Entropy (BMGE) (Criscuolo and Gribaldo, 2010). Partial sequences identified were ignored from the final analyses. All the alignments are available upon request. Phylogenetic analyses were carried out by maximum likelihood approach using IQTree2 (Minh et al., 2020). The IQTree Model finder was used to choose the best-fit models for all the analyses (Kalyaanamoorthy et al., 2017). Ultrafast bootstrapping (-B -N 1000) and non-parametric bootstrapping (-b -N 1000) support values were generated with IQTree2. FigTree v1.4.4 (http://tree.bio.ed.ac.uk/software/figtree/) was used to visualize and analyze the phylogenetic data.

### Cell culture and cultivation

The *T. vaginalis* strain Tv17-48 was used for this study (Kulda et al., 1982). The cells were axenically cultured at 37°C in tryptone–yeast extract–maltose (TYM) medium at pH 6.2 with 10% heat-inactivated horse serum. For the growth and selection of co-transfected cells, the TYM medium was supplemented with 100 mg/ml of geneticin and 40 mg/ml of puromycin.

### Expression vector construction and nucleofection into the *T. vaginalis*

VPS26A/B (TVAG_160820) and VPS26C (TVAG_332210) were expressed in *T. vaginalis* with a C-terminal HA tag using the vector pTagVag-HA-Neo (Hrdy et al., 2004). Both these gene paralogs chosen for expression of retromer and retriever complex proteins showed the highest expression (Gould et al., 2013) and highest sequence identity with human ortholog proteins (Fig. S5). The genes were expressed under the control of their respective native promoters of 250 bp upstream of the coding sequence. However, for VPS29, the highest-expressing paralog failed cloning, so we chose another paralog with similar conservation of functional residues that is also known to be natively expressed by the cell. VPS29 (TVAG_378930) was cloned with C-terminal V5 tag using vector pTagVag-V5-Pur (Štáfková et al., 2018). VPS29 expression was under the control of the native promoter of 300 bp upstream. VPS26A/B (TVAG_160820) was re-cloned with the C-terminal V5 tag for co-expression with VPS26C. The genes were amplified by PCR using genomic DNA of Tv17-48 as a template and cloned in the vectors via SacII and BamHI restriction sites. The vectors were co-transfected into the Tv17-48 trichomonad cells by nucleofection using the Human T cell Nucleofector Kit (Lonza) according to Zimmann et al. (2022) and Janssen et al. (2018). The gene sequence and PCR primers are provided in Table S7.

### Immunofluorescence microscopy and image analysis

*T. vaginalis* transfected cells were harvested by centrifugation at 1200 ***g*** for 10 min. The cells were then washed with PBS and fixed using 2% formaldehyde as described by Woessner and Dawson (2012). The fixed cells attached to poly L-lysine-treated coverslips were incubated overnight at 4°C with the following primary antibodies: rabbit polyclonal anti-HA antibody (Santa Cruz Biotechnology Inc., cat. no. sc805, 1:1000) and mouse monoclonal anti-V-5 antibody (Thermo Fisher Scientific, cat. no. 37-7500, 1:1000). Alexa Fluor 594 donkey anti-rabbit-IgG (Life Technologies,cat. no. A11012, 1:1000) and Alexa Fluor 488 donkey anti-mouse-IgG (Life Technologies, cat. no. A-21202, 1:1000) were used as secondary antibodies. The nucleus was stained with 4′,6-diamidino-2-phenylindole (DAPI). Slides were observed using a Leica TCS SP8 confocal laser scanning microscope with a 63× oil immersion objective. Captured images were deconvolved using Huygens Professional v19.10 (https://svi.nl/Huygens-Professional) and processed using ImageJ/Fiji open-source software (Schindelin et al., 2012). Colocalization analysis was performed using a voxel-based colocalization analysis tool in Huygens Professional v19.10 in *z*-stacks to calculate Pearson's correlation coefficient using Costes method.

### Structural analysis

PDB format files were obtained from AlphaFold (Jumper et al., 2021; Varadi et al., 2022) for *T. vaginalis* and from the Protein Data Bank server for Human retromer and retriever proteins. *T. vaginalis* AlphaFold structures were used to collect the secondary structures and to align them with the multiple sequence alignments tool using ESPript 3.0 (Robert and Gouet, 2014) (Fig. S5). Tertiary structures of the *T. vaginalis* retromer and retriever structures predicted by AlphaFold were aligned with 3D crystal structures of human trimeric complexes using TM-align (Zhang, 2005) to confirm the quality of confidence of each structural alignments, and then superimposed in PyMOL 3.0 (https://pymol.org/) using the ‘align’ command to predict the topology of both the trimeric complexes in *T. vaginalis*. Superimposed images of the human and *T. vaginalis* trimers were obtained from PyMOL 3.0 (Fig. 5).

## Supplementary Material

10.1242/joces.261949_sup1 Supplementary information

Table S1. List of genomic databases for Parabasalids and Anaeramoeba subjected in this study.

Table S2. List with identification numbers for all the identified Retromer complex proteins and its cargoes from Homology searches.

Table S3. List with identification numbers for all the identified Retriever complex proteins and its cargoes.

Table S4. List of all the identified CCC complex proteins in this study

Table S5. List of all the identified WASH complex proteins.

Table S6. List of all the identified Sorting nexins in this study.

Table S7. List of sequences and primers used for expression of selected protein candidates

Table S8. Transcriptomic and proteomic sources of evidence for expression of Retromer and Retriever trimeric protein paralogs identified in T. vaginalis

Table S9. TM-align scores calculated for quantitative comparison of Human known structures with predicted T. vaginalis structures.
