# Supplementary material for: The retromer and retriever systems are conserved and differentially expanded in parabasalids
Source: J Cell Sci. 2024 Jul 12;137(13):jcs261949. doi: 10.1242/jcs.261949 (PMC11267458; doi:10.1242/jcs.261949)
Supplement: Supplementary information [file joces-137-261949-s1.pdf]

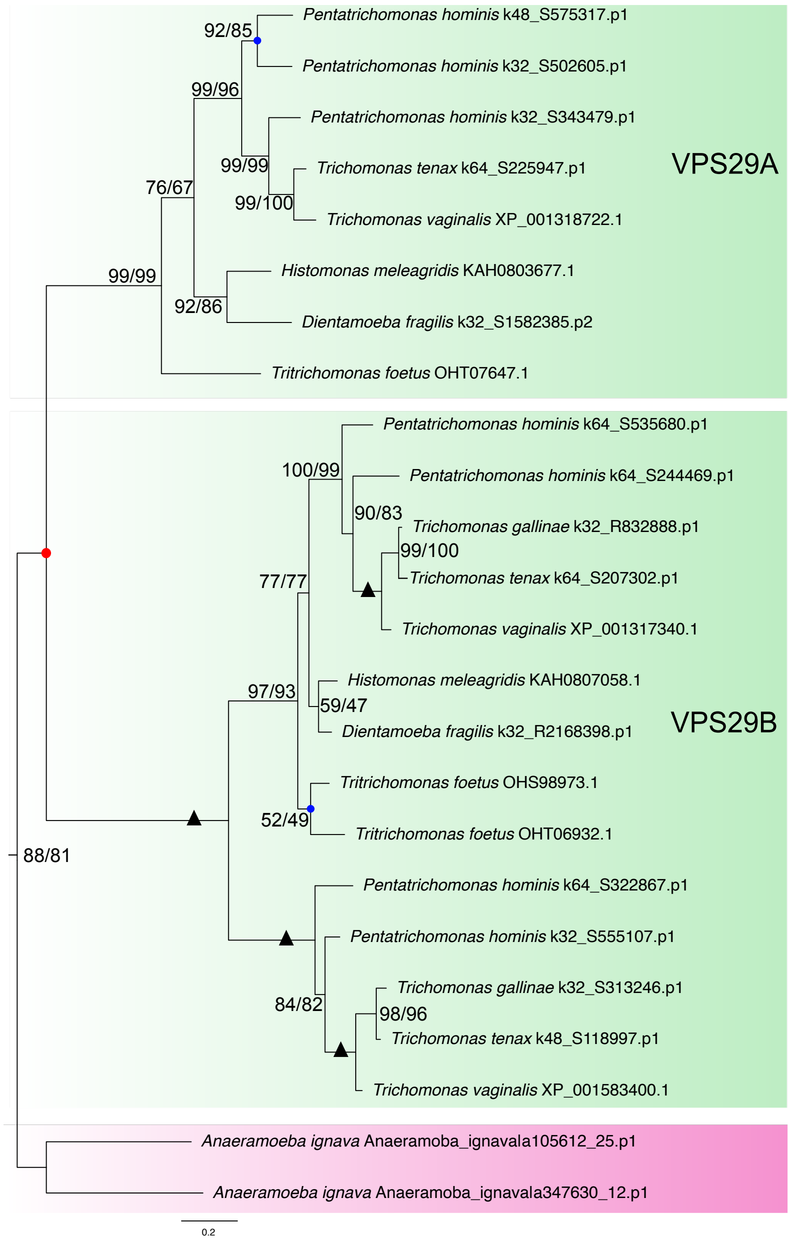

Fig. S1. Maximum likelihood phylogenetic analysis tree was constructed using IQTree (Best fit: Q.yeast+G4) with 24 sequences and 180 sites for VPS29 expansion events in parasitic Parabasalids. This phylogenetic tree includes all the orthologues identified from homology searches in Parabasalids and *A. ignava*. The clades in green represents orthologues of VPS29 identified in the parasitic Parabasalia lineage. The clade in pink represents paralogues identified in *A. ignava* where the tree is rooted. A partial sequence of VPS29 from *A. ignava* was eliminated from this analysis for the robustness of the dataset. The red dot represents an ancestral duplication event during the speciation of the parasitic Parabasalids and the blue dots represent species-specific duplication events. Support values for each node are presented in the format of UFB/NP. Branches with support values of 100 for both ultrafast and NP bootstrapping are represented with black solid triangles.

Journal of Cell Science • Supplementary information

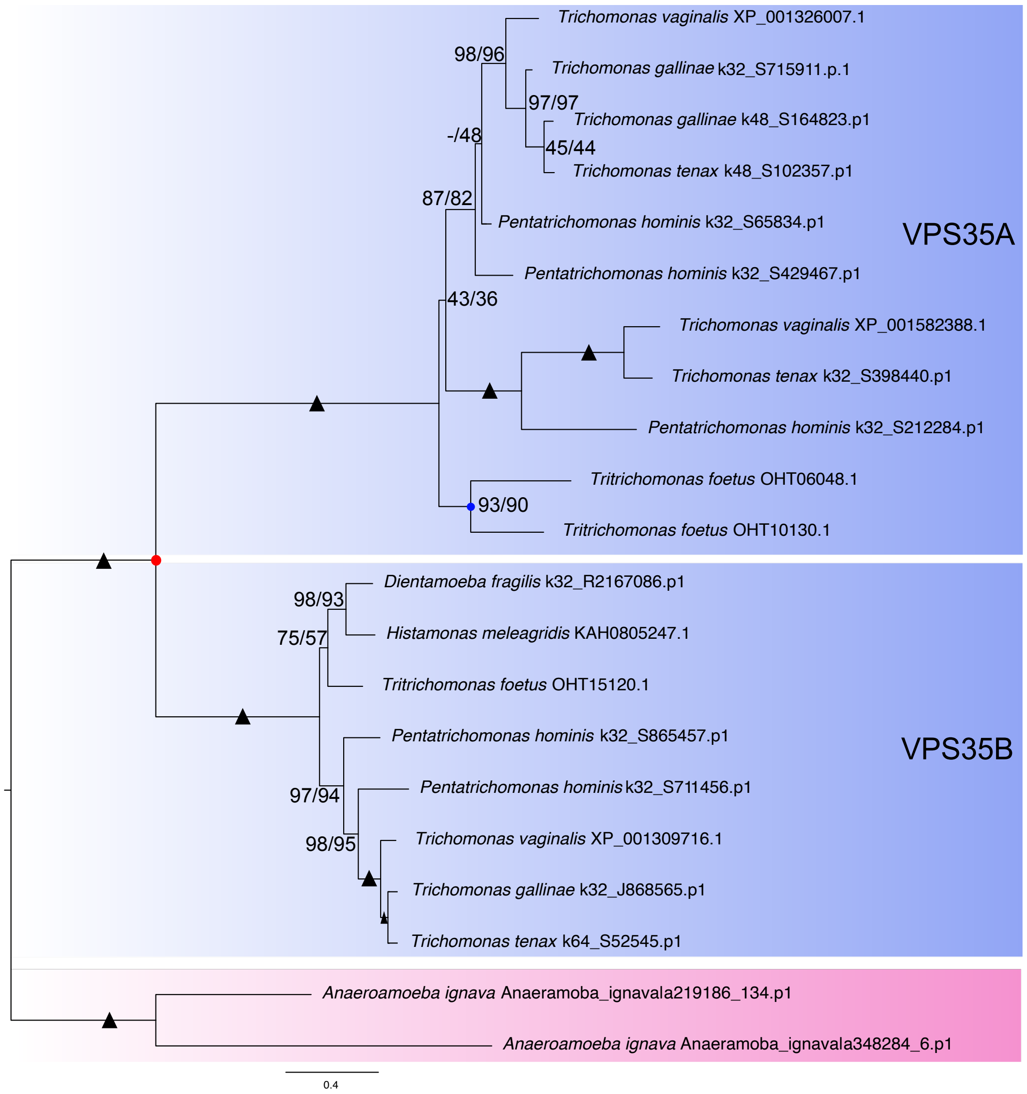

Fig. S2. Maximum likelihood phylogenetic analysis tree was constructed using IQTree (Best fit: Q.yeast+I+G4) with 21 sequences and 545 sites for VPS35 expansion events in parasitic Parabasalids. This phylogenetic tree includes all the orthologues identified from homology searches in Parabasalia and *A. ignava*. This tree was rooted at mid-point. The clades in blue represents orthologues of VPS35 identified in the parasitic Parabasalia lineage. The clade in pink represents paralogues identified in *A. ignava*. Two partial sequences of VPS35 from *A. ignava* were eliminated from this analysis to maintain the robustness of the dataset. The red dot represents an ancestral duplication event during the speciation of the parasitic Parabasalids and the blue dots represent species specific duplication events. Support values for each node are presented in the format of UFB/NP. Branches with support values of 100 for both ultrafast and NP bootstrapping are represented with black solid triangles.

Journal of Cell Science • Supplementary information

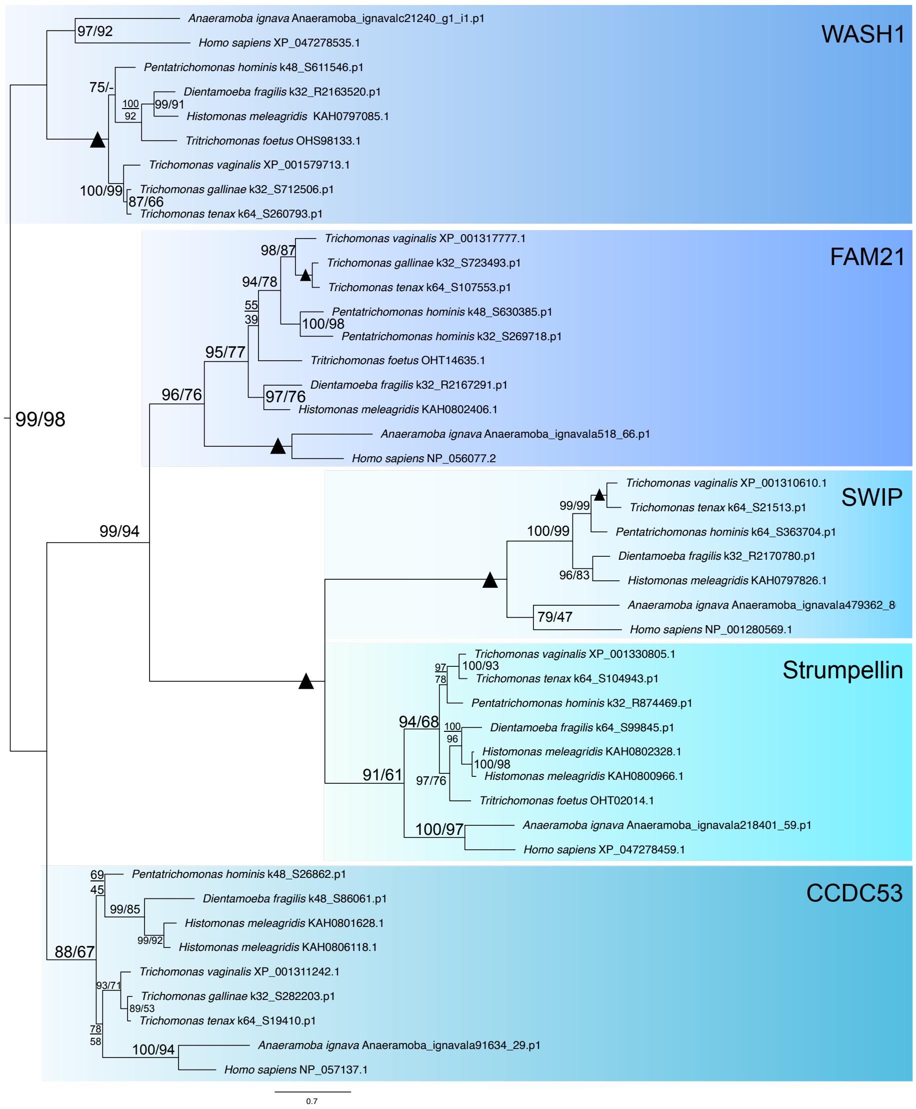

Fig. S3. Maximum likelihood phylogenetic analysis tree was constructed using IQTree (Best fit: LG+F+G4) with 44 sequences and 424 sites for the WASH complex in the parasitic Parabasalids and *A. ignava*. The phylogenetic tree was constructed using all the WASH complex protein homologues and orthologues identified from Comparative genomic analysis (Figure 4). WASH complex protein sequences from *H. sapiens* were used as reference for the phylogenetic characterization of each WASH complex protein. The tree was rooted at WASH1. Support values for each node are depicted in the format UFB/NP. Branches with support values of 100 for both ultrafast and NP bootstrapping are represented with black solid triangles.

Journal of Cell Science • Supplementary information

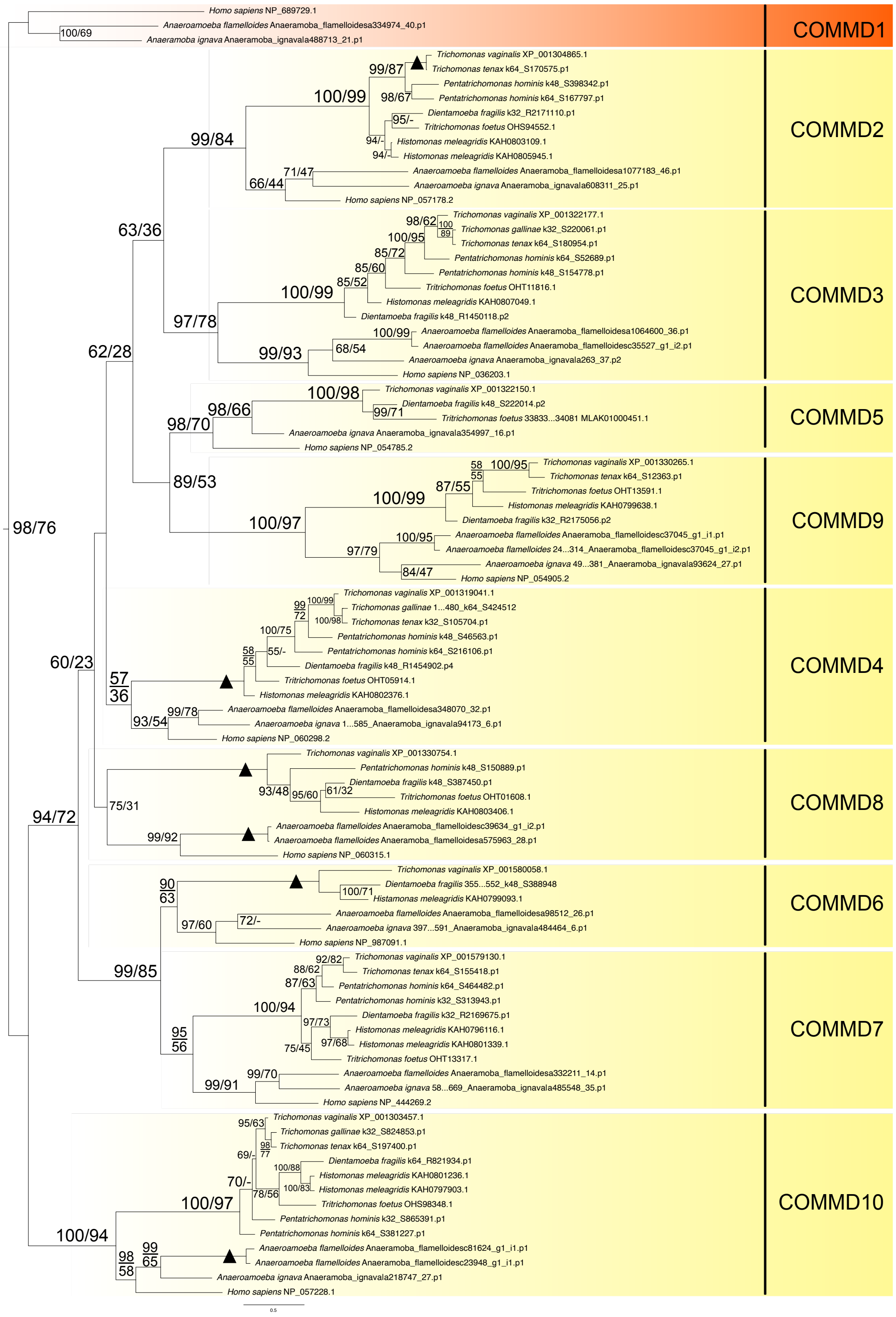

Journal of Cell Science • Supplementary information

Fig. S4. Maximum likelihood phylogenetic analysis tree was constructed using IQTree (Best fit: LG+F+G4) with 89 sequences and 137 sites for characterization of COMMD1-10 proteins of the CCC complex in Parabasalia and Anaeramoeba lineages. The phylogenetic tree was constructed using all the COMMD protein homologues and orthologues identified from Comparative genomic analysis (Fig. 5). COMMD protein sequences from *H. sapiens* were used as reference for the phylogenetic characterization of each COMMD protein. The tree was rooted at COMMD1 clade (represented in red) and COMMD2-10 clades are represented in yellow. Support values for each node are depicted in the format UFB/NP. Branches with support values of 100 for both ultrafast and NP bootstrapping are represented with black solid triangles.

Journal of Cell Science • Supplementary information

A

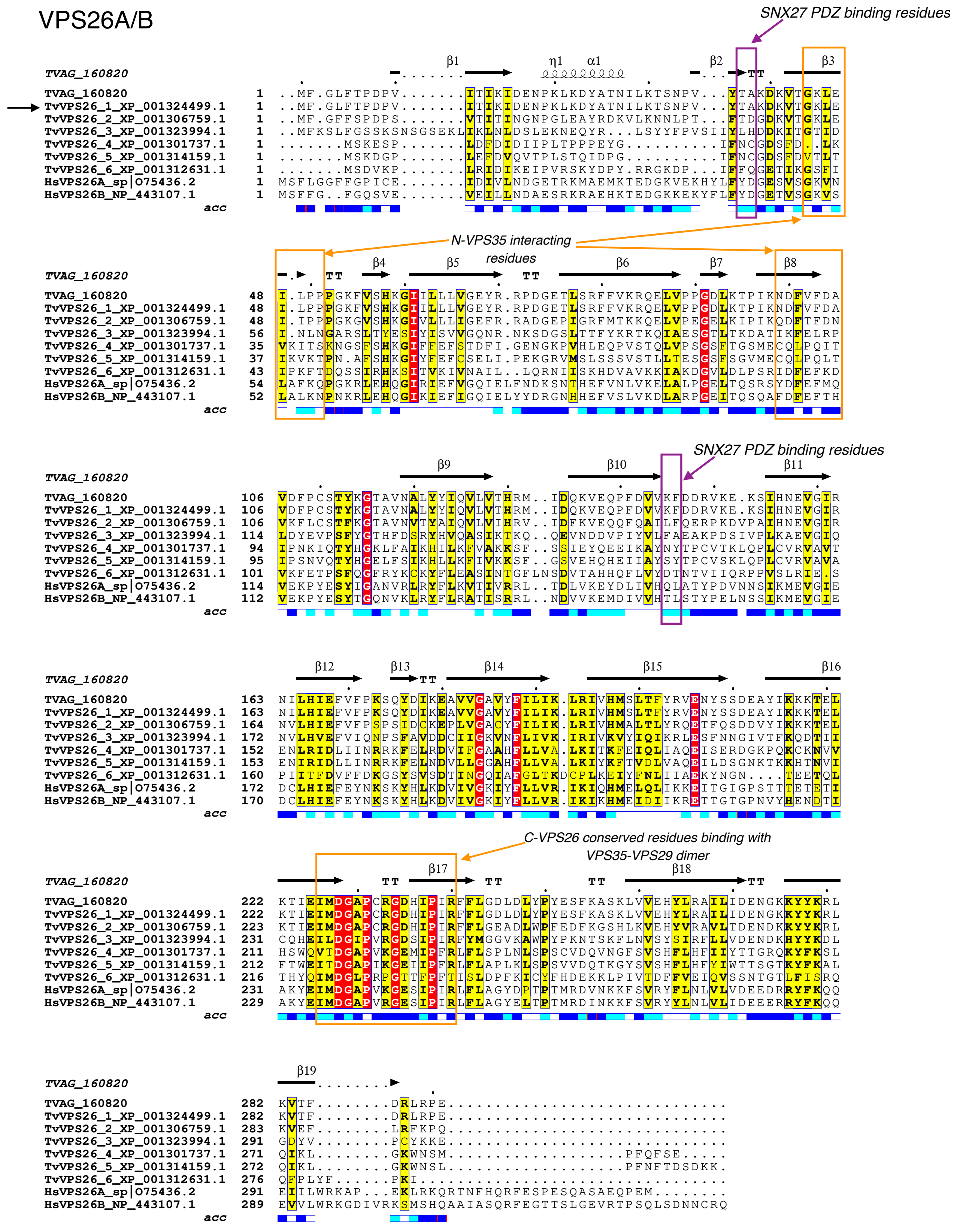

Journal of Cell Science • Supplementary information

B

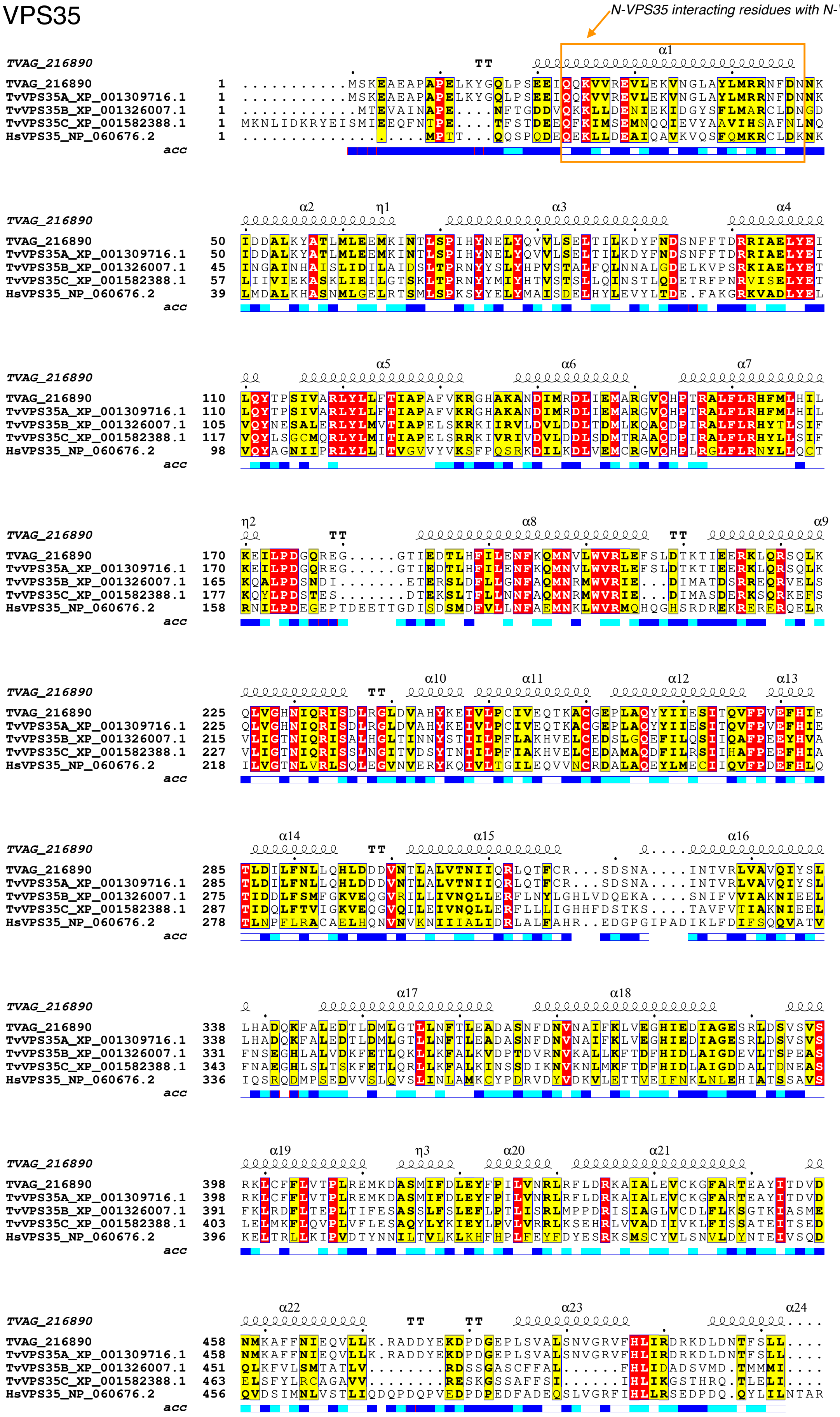

Journal of Cell Science • Supplementary information

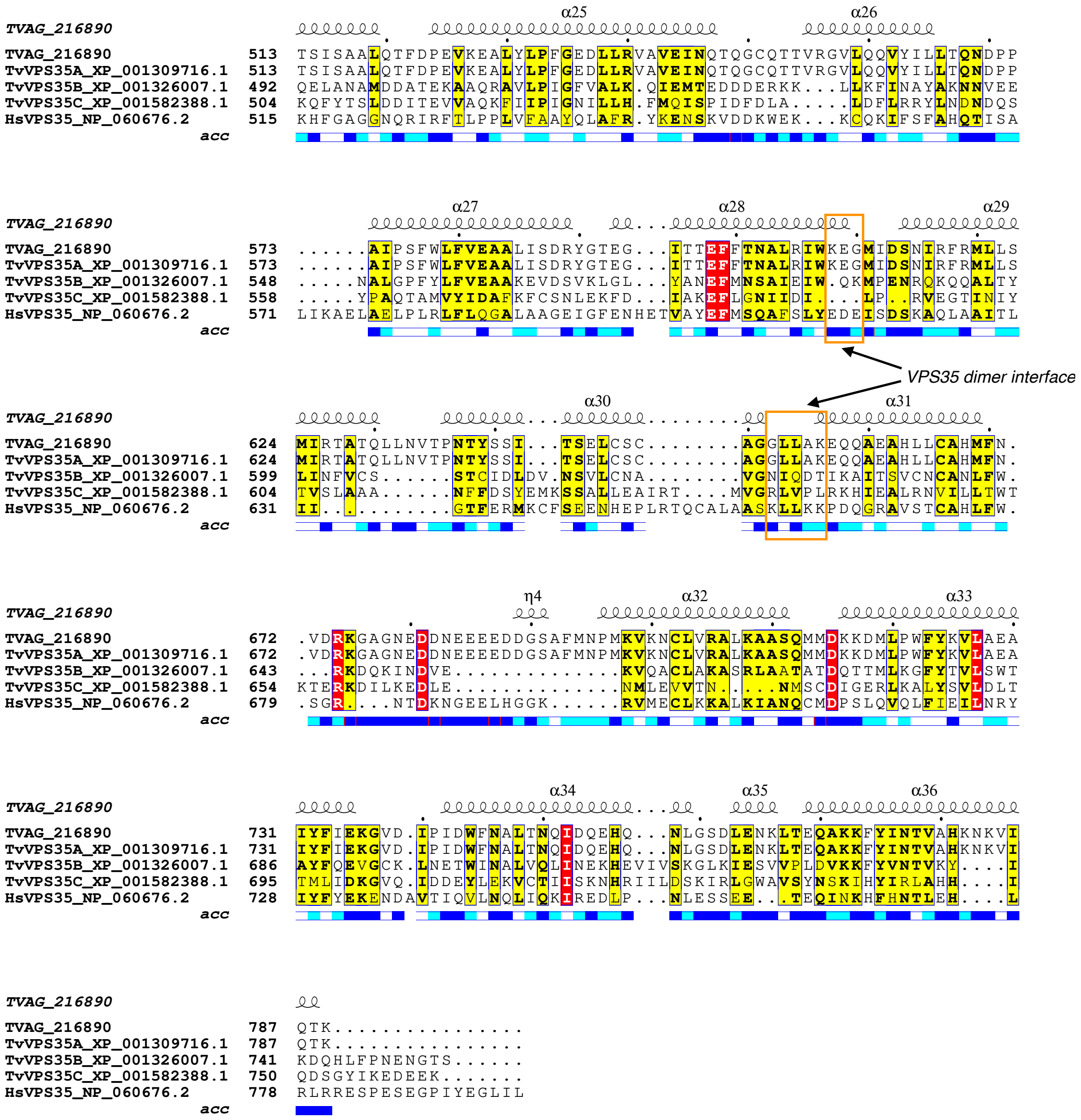
TVAG_216890
TVAG_216890
TvVPS35A_XP_001309716.1
TvVPS35B_XP_001326007.1
TvVPS35C_XP_001582388.1
HsVPS35_NP_060676.2
acc
α25
α26
α27
α28
α29
α30
α31
α32
α33
α34
α35
α36
η4
VPS35 dimer interface

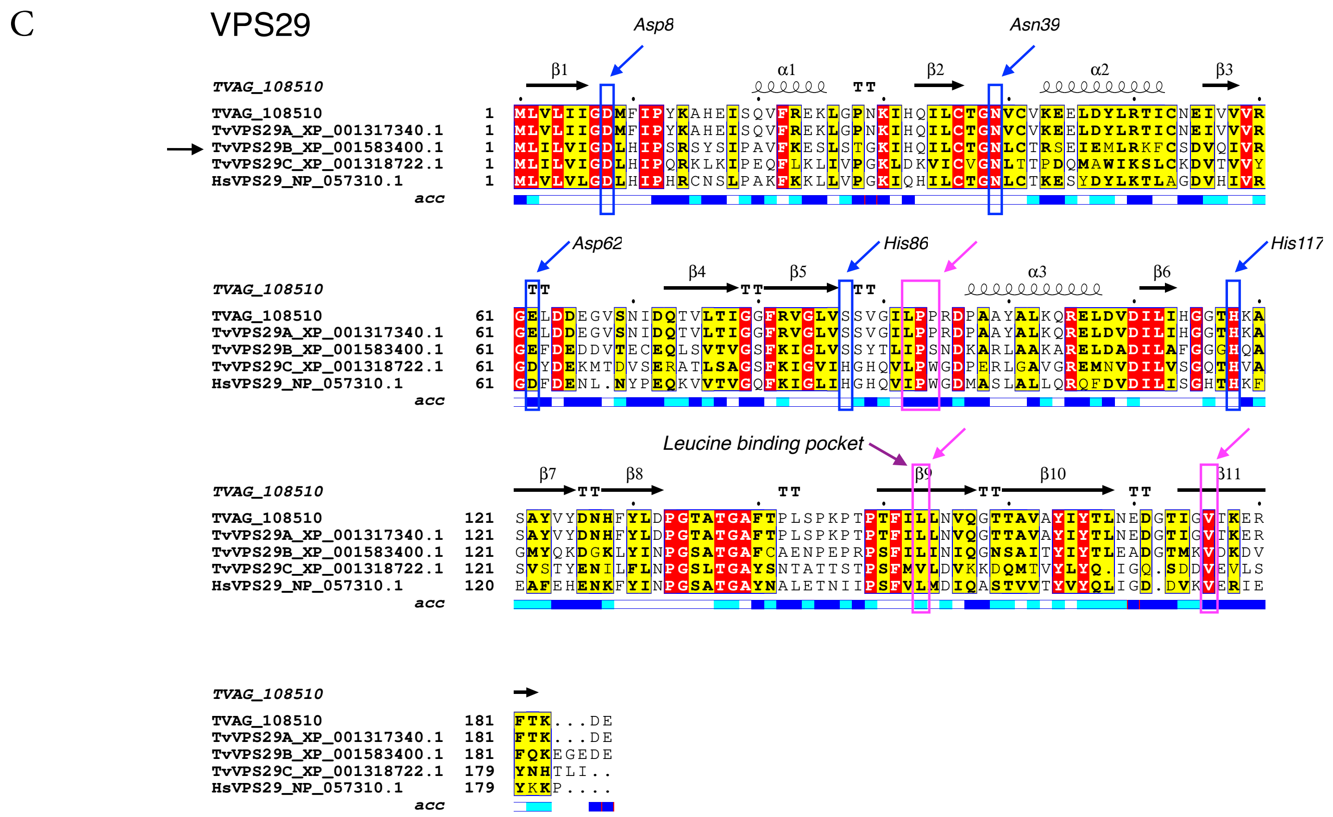

Key : Metallo-phosphoesterase fold residues marked with a blue arrow ( ) and blue box

Key : VPS35-like interacting residues marked with a magenta arrow ( ) and magenta box

Journal of Cell Science • Supplementary information

VPS26C

D

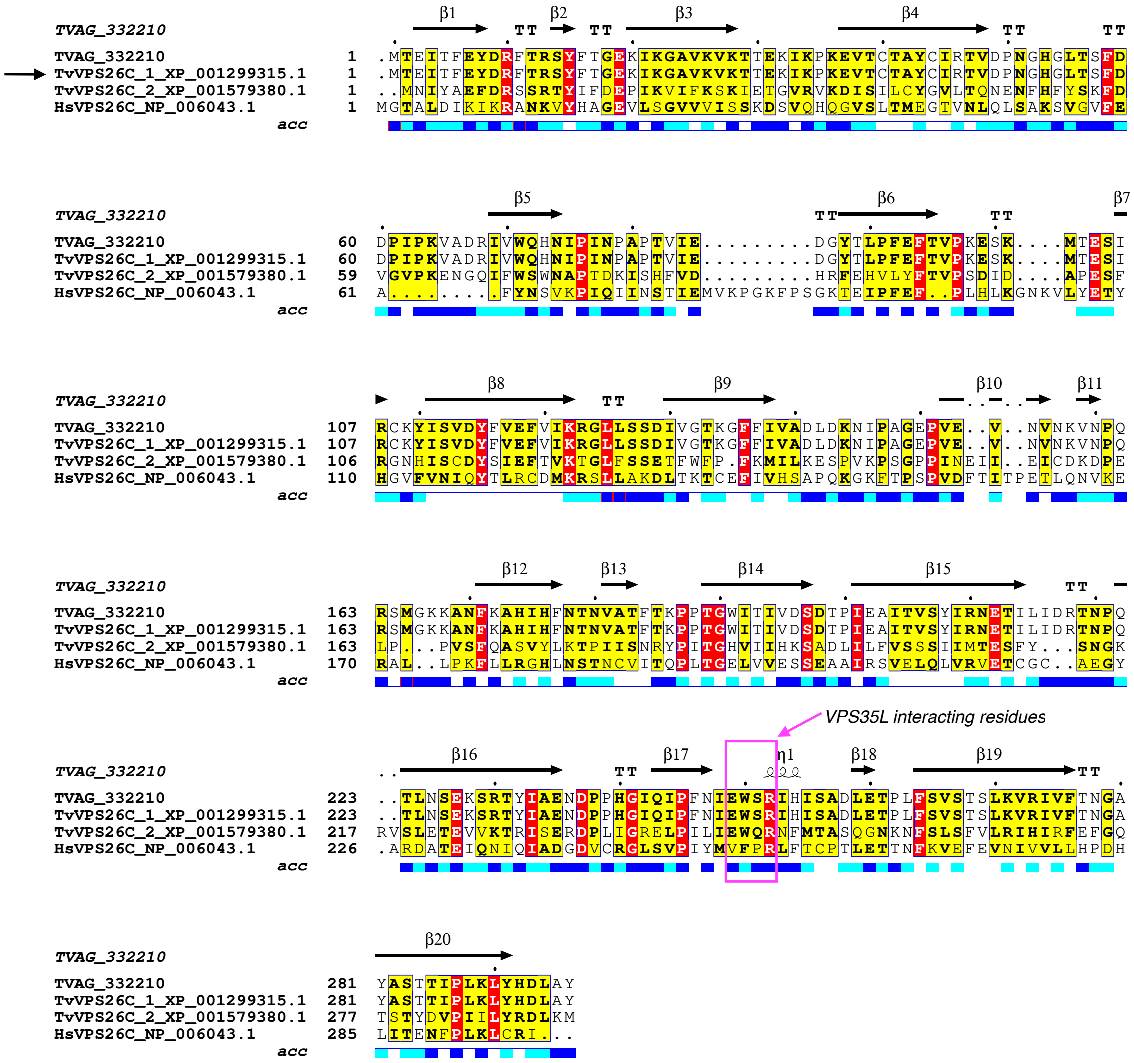

Journal of Cell Science • Supplementary information

E

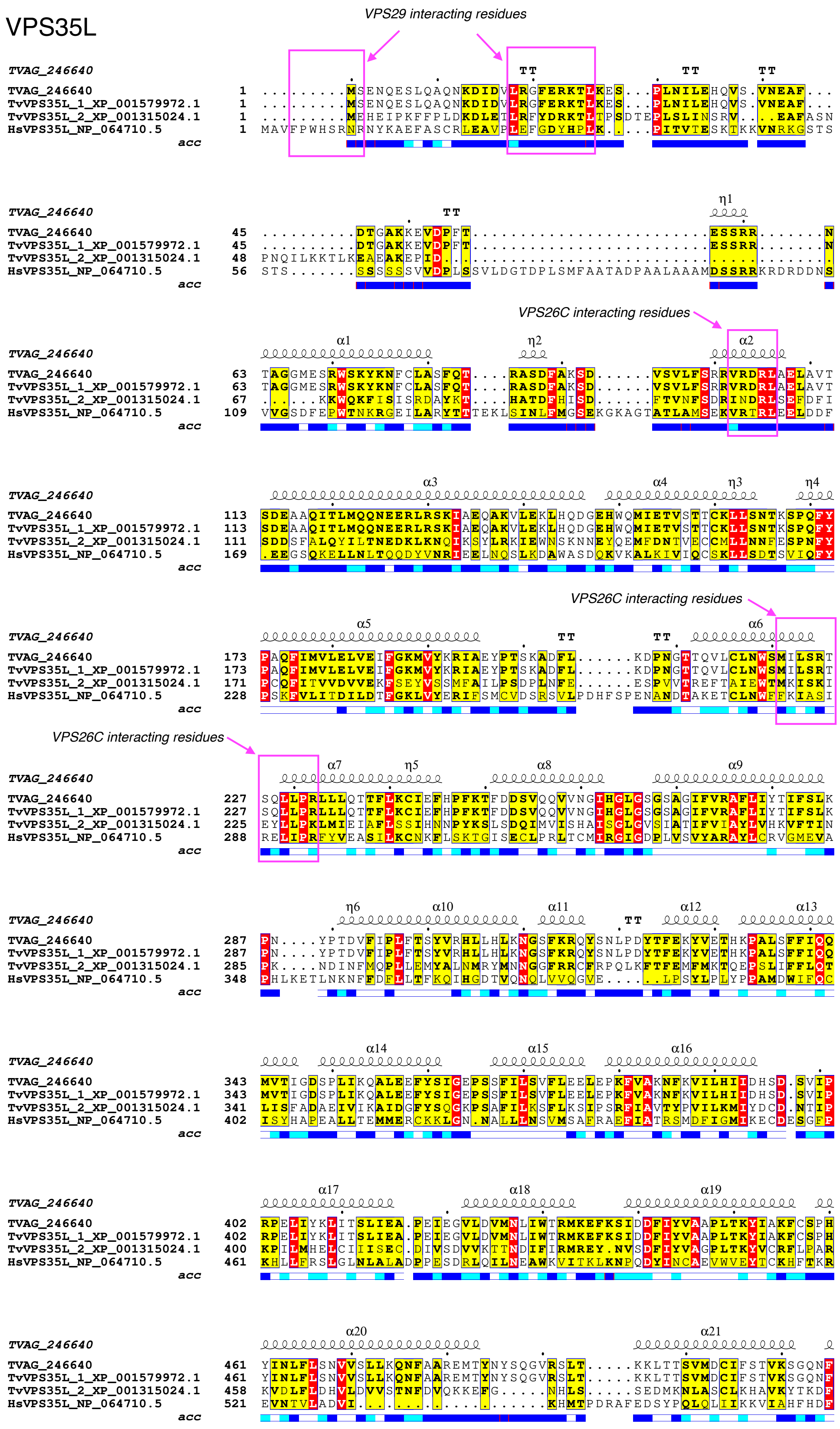

Journal of Cell Science • Supplementary information

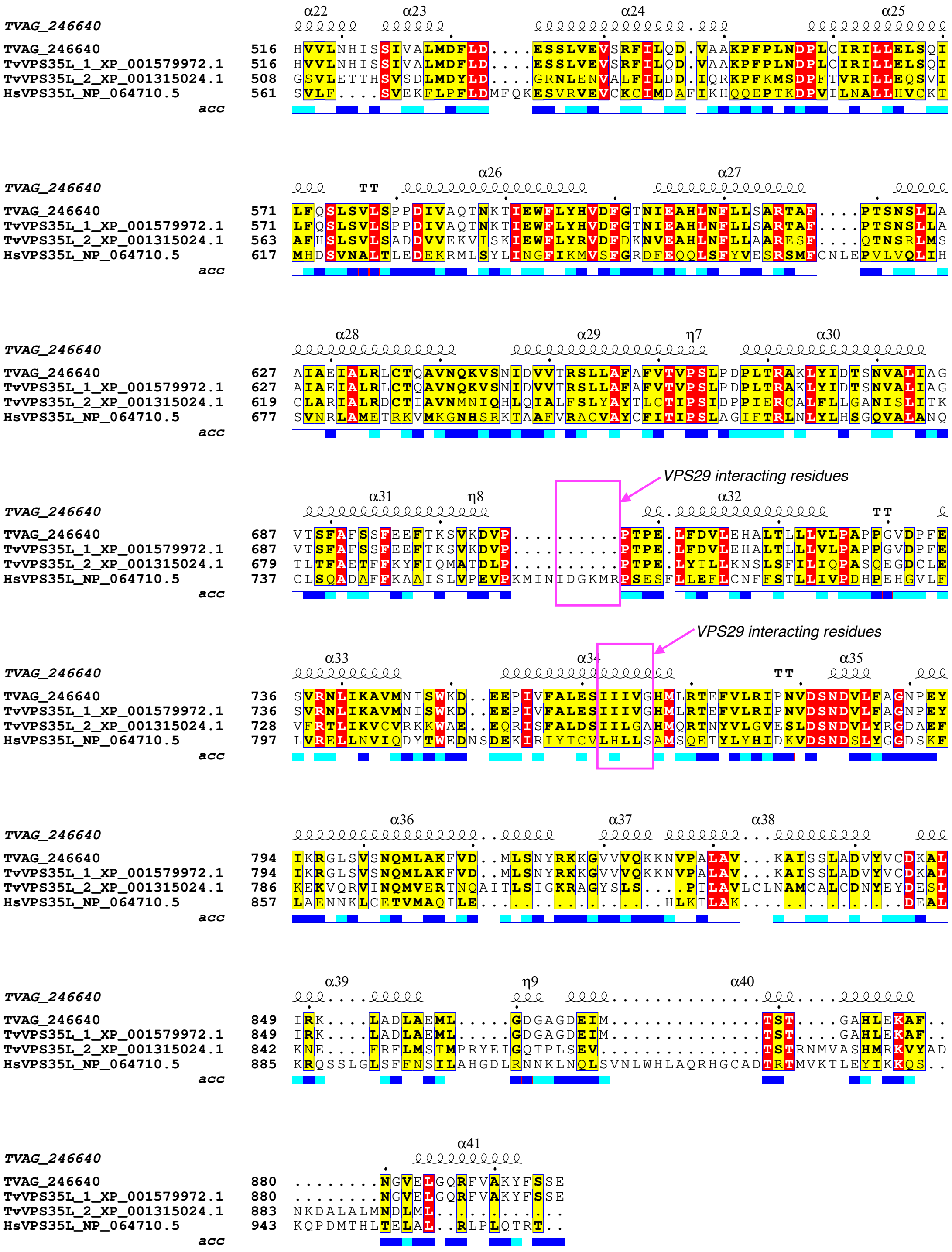

Journal of Cell Science • Supplementary information

Fig. S5. ESPript 3.0 output of multiple sequence alignment (MSA) for each identified Retromer and Retriever complex proteins in *T. vaginalis*. Five panels represented alphabetically for MSA of VPS26A/B (A), VPS35 (B), VPS29 (C), VPS26C (D), VPS35L (E) of all the identified *T. vaginalis* paralogs with their Human homologs. Secondary structure elements of highest expressing paralog are displayed above the MSA. Secondary structure elements are labelled as coiled squiggles depicting helices, arrows depicting β- strands, and TT letters for turns in the protein structure. Below the MSA is the bar of solvent accessibility for the protein, blue, white, and cyan represents accessible, buried, and intermediate residues respectively. Similar residues are highlighted in yellow and identical residues are highlighted in red. All the proteins description are labelled before the NCBI accession numbers in the MSAs. The secondary structure for each *T. vaginalis* protein is chosen based on highest sequence conservation with known Human proteins amongst all the identified paralogs of a protein in *T. vaginalis*. Protein paralogs chosen for experiments are labelled with a black arrow. Annotations refering to functional residues in the sequence alignments are made with appropriate keys where necessary.

Table S1. List of genomic databases for Parabasalids and Anaeramoeba subjected in this study.

Available for download at
https://journals.biologists.com/jcs/article-lookup/doi/10.1242/jcs.261949#supplementary-data

Table S2. List with identification numbers for all the identified Retromer complex proteins and its cargoes from Homology searches.

Available for download at
https://journals.biologists.com/jcs/article-lookup/doi/10.1242/jcs.261949#supplementary-data

Journal of Cell Science • Supplementary information

Table S3. List with identification numbers for all the identified Retriever complex proteins and its cargoes.

Available for download at
https://journals.biologists.com/jcs/article-lookup/doi/10.1242/jcs.261949#supplementary-data

Table S4. List of all the identified CCC complex proteins in this study

Available for download at
https://journals.biologists.com/jcs/article-lookup/doi/10.1242/jcs.261949#supplementary-data

Table S5. List of all the identified WASH complex proteins.

Available for download at
https://journals.biologists.com/jcs/article-lookup/doi/10.1242/jcs.261949#supplementary-data

Table S6. List of all the identified Sorting nexins in this study.

Available for download at
https://journals.biologists.com/jcs/article-lookup/doi/10.1242/jcs.261949#supplementary-data

Table S7. List of sequences and primers used for expression of selected protein candidates

Available for download at
https://journals.biologists.com/jcs/article-lookup/doi/10.1242/jcs.261949#supplementary-data

Journal of Cell Science • Supplementary information

Table S8. Transcriptomic and proteomic sources of evidence for expression of Retromer and Retriever trimeric protein paralogs identified in T. vaginalis

Available for download at
https://journals.biologists.com/jcs/article-lookup/doi/10.1242/jcs.261949#supplementary-data

Table S9. TM-align scores calculated for quantitative comparison of Human known structures with predicted T. vaginalis structures.

Available for download at
https://journals.biologists.com/jcs/article-lookup/doi/10.1242/jcs.261949#supplementary-data
